# Transcriptional Enhancers in Protein-Coding Exons of Vertebrate Developmental Genes

**DOI:** 10.1371/journal.pone.0035202

**Published:** 2012-05-02

**Authors:** Deborah I. Ritter, Zhiqiang Dong, Su Guo, Jeffrey H. Chuang

**Affiliations:** 1 Department of Biology, Boston College, Chestnut Hill, Massachusetts, United States of America; 2 Department of Bioengineering and Therapeutic Sciences, Programs in Biological Sciences and Human Genetics, University of California San Francisco, San Francisco, California, United States of America; Ecole Normale Supérieure de Lyon, France

## Abstract

Many conserved noncoding sequences function as transcriptional enhancers that regulate gene expression. Here, we report that protein-coding DNA also frequently contains enhancers functioning at the transcriptional level. We tested the enhancer activity of 31 protein-coding exons, which we chose based on strong sequence conservation between zebrafish and human, and occurrence in developmental genes, using a Tol2 transposable GFP reporter assay in zebrafish. For each exon we measured GFP expression in hundreds of embryos in 10 anatomies via a novel system that implements the voice-recognition capabilities of a cellular phone. We find that 24/31 (77%) exons drive GFP expression compared to a minimal promoter control, and 14/24 are anatomy-specific (expression in four anatomies or less). GFP expression driven by these coding enhancers frequently overlaps the anatomies where the host gene is expressed (60%), suggesting self-regulation. Highly conserved coding sequences and highly conserved noncoding sequences do not significantly differ in enhancer activity (coding: 24/31 vs. noncoding: 105/147) or tissue-specificity (coding: 14/24 vs. noncoding: 50/105). Furthermore, coding and noncoding enhancers display similar levels of the enhancer-related histone modification H3K4me1 (coding: 9/24 vs noncoding: 34/81). Meanwhile, coding enhancers are over three times as likely to contain an H3K4me1 mark as other exons of the host gene. Our work suggests that developmental transcriptional enhancers do not discriminate between coding and noncoding DNA and reveals widespread dual functions in protein-coding DNA.

## Introduction

The functions in a genome are often conceptually divided into protein functions for coding DNA and regulatory functions for noncoding DNA. This division is based on the intuition that constraints associated with encoding a protein would prevent the evolution of noncoding functions in a coding region. However, the validity of this division has not been well-studied. One important class of regulatory functional elements in noncoding DNA is enhancers. These are DNA sequences classically found distal to gene promoters and associated with tissue- or temporally-specific transcriptional regulation of gene expression, especially for developmental genes [1]–[8]. Here we investigate whether protein-coding DNA can contain enhancer functions similar to those found in noncoding DNA.

Prior computational and evolutionary studies at the motif level have shown that coding DNA can hold noncoding information. This ability to contain other functional information arises from the redundancy of synonymous codons. For example, Itzkovitz and Alon compared the human genetic code to alternative permuted codes, finding that the genetic code is nearly ideal for containing short functional motifs within protein-coding DNA [9]. Using a sequence conservation approach, hundreds of unusually conserved nucleotide motifs have been found in coding sequences even after correcting for protein-level constraint [10], [11]. Additionally, multiple genome-wide transcription factor and histone modification studies have reported low-levels of protein binding within coding sequence [12]–[14]. However, because a substantial fraction of protein-DNA binding is believed to be neutral, it has often assumed that such binding in coding regions is non-functional [15]. In any case, assessments of functional motifs in coding sequence do not strongly test the ability of protein coding sequence to hold dual functions. This is because motifs are short in comparison to mRNA lengths.

Protein-coding sequences can be more critically tested by considering developmental enhancer activity. Developmental enhancers are typically much longer than individual TF-binding motifs and are often associated with strong sequence constraint. Highly conserved noncoding sequences have shown frequent enhancer activity in developmental expression assays [5], [7], [16]. For example, three-fourths of noncoding sequences with >60% human-teleost conservation have shown enhancer activity in developmental assays [3]. Therefore discovery of developmental enhancers in coding regions would indicate that long, highly constrained regulatory functions can evolve in coding regions despite the protein-coding constraint.

Relatively little is known about enhancers in coding sequence. Coding exon-controlled enhancer activity has been reported in a few cell line experiments, e.g. from the APOE, ADAMTS5 and BCL-2 genes [17]–[19], but whole embryo experiments would provide more definitive evidence of developmental activity. Such embryonic data is relatively sparse and has shown conflicting results. Tumpel et al observed that the second exon of *Hoxa2* contains a consistent but weak developmental enhancer within a coding region, though reporter activity was found to be stronger when the coding region was combined with an adjacent noncoding sequence [20]. Similarly, Lampe et al identified a coding enhancer in the first exon of Hoxa2 [21]. However, Woolfe et al tested three coding sequences (from Sox21, Pax6 and SHH in zebrafish developmental assay), but found little to no expression [5]. The relative dearth of experimental data has made it unclear how prevalent coding developmental enhancers are.

To address this question, we investigated the enhancer functions of 31 coding sequences from a variety of developmental genes orthologous between human and zebrafish. We chose **C**onserved **C**oding **E**lements (hereafter CCEs) with strong conservation across vertebrate species for this study, as we expected these might be more likely to contain enhancers [22]. Using whole-embryo experiments, we found that the coding sequence of many developmental genes contains enhancers that drive tissue-specific gene expression. Our results indicate that enhancers in coding regions and in noncoding regions have similar levels of activity, tissue-specificity and enhancer-associated histone modifications. Thus the protein-coding constraint does not exclude noncoding developmental regulatory information. Our work indicates that complex additional functions may be commonly harbored in protein-coding sequences of vertebrate genomes.

## Results

### Conserved Coding Elements Act as Enhancers

Conserved Coding Elements (CCEs) were identified using minimal criteria of >60% DNA sequence conservation between zebrafish and human, 100–1000 bp length, and occurrence within a set of developmental genes orthologous between zebrafish and human. These criteria were chosen to be similar to those used for identifying Conserved Noncoding Elements (CNEs) in a previous study of CNE enhancer activity [3] to allow for comparison of CCEs and CNEs. CCEs meeting these criteria were refined to a set of 31 for experimental testing with a range of conservation levels and exon ranks (Data File S1 and Data File S4). 26 CCEs corresponded to a complete zebrafish coding exon. The remaining 5 CCEs were Ultra Conserved Regions (UCRs) from the zebrafish genome identified originally in Bejerano et al [1]. Each of these UCRs exhibited partial overlap with a zebrafish coding exon. The zebrafish-human conservation levels of the tested sequences ranged from 67%–95% (avg. 79%). In comparison, zebrafish coding exons have on average only 48% similarity with the human sequence, and UTRs are only 9% similar. The test set has stronger silent site conservation than other coding sequences as well (50% 4-fold conservation vs. 35% for random exons) [23], [24].

We sub-cloned each CCE into a Tol2 vector, upstream of an E1B minimal promoter driving EGFP (see Methods and Figure S1). For each CCE, ∼150–300 zebrafish embryos were injected at the 1-cell stage with the vector and transposase mRNA. A control vector containing the minimal promoter upstream of EGFP but lacking an insert sequence was also assayed to assess the expression of EGFP under the minimal promoter only.

**Figure 1:**
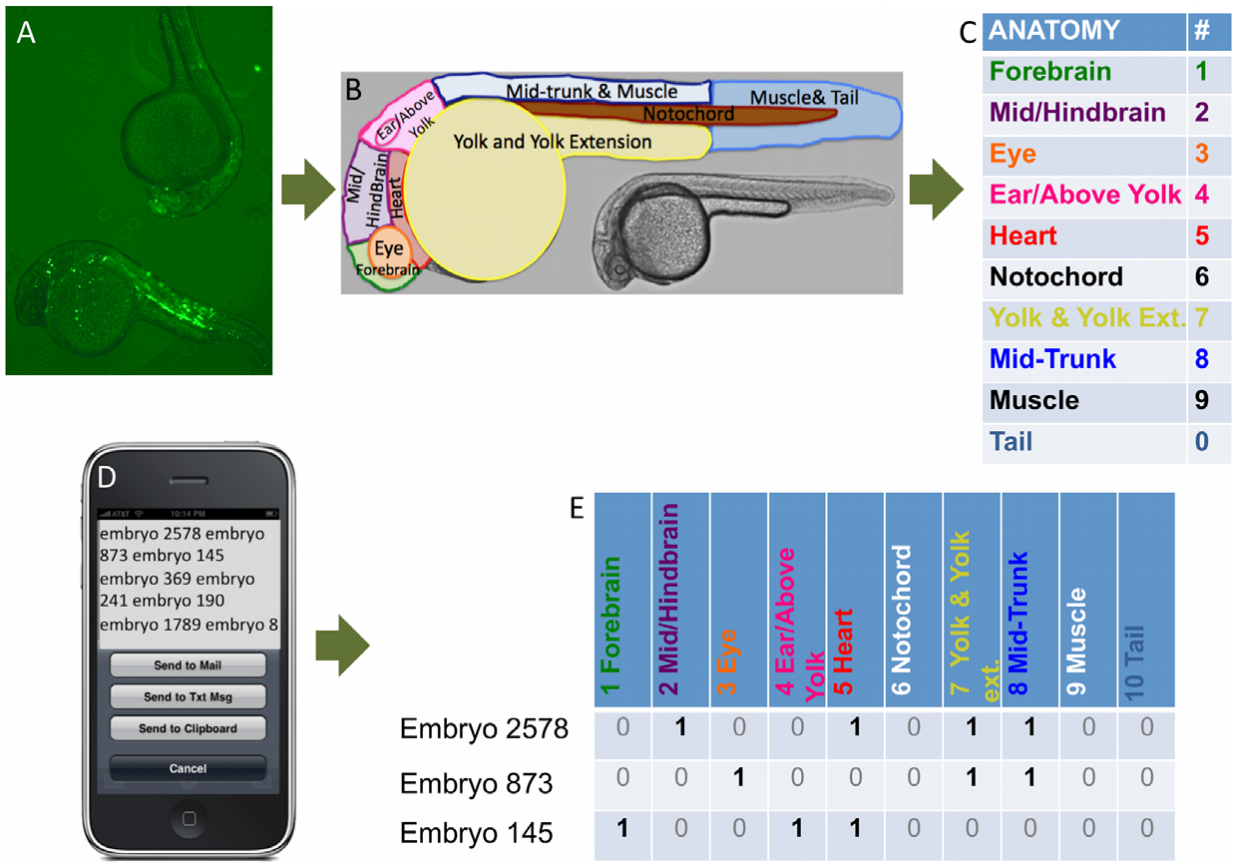
Overview of EGFP expression scoring process. (*A*) Zebrafish with eGFP expression are scored using a (*B*) limited anatomy corresponding to (*C*) numerical values. (*D*) These are interpreted using the iPhone app Dragon Dictation. (*E*) A PERL script transforms text into numerical strings representing embryo expression in each anatomy. These data are analyzed to determine anatomical regions with significant expression for each CCE via a proportions test and a Wilcoxon rank-sum test (see Methods and Supplementary Data File S2).

Embryos were scored for EGFP transient expression at 22–30 hours in 10 anatomies. Transient expression of reporter genes in zebrafish has been successfully used to identify noncoding enhancers by several groups [3], [5], [8], [25]–[31]. Such transient expression assessed over large numbers of embryos has been found to yield enhancer assessments consistent with stable transgenics [5], [8], [26]. To facilitate data input and analysis, we developed a novel expression scoring technique that uses the voice recognition capabilities of a cellular phone and custom PERL scripts to assess significant expression of experimental constructs compared to the control (see Methods and Figure 1). This technique allows for simultaneous assessment of several dozen embryos in each microscope viewing, resulting in increased numbers of scored embryos and improved quantification of expression.

We identified CCE enhancers by comparing the fraction of embryos with activity driven by the CCE to the fraction of embryos with activity driven by the control, on an anatomy-specific basis (see Methods, Figure S2). Thresholds were based on a proportions test and non-parametric Wilcoxon test, and anatomies with significant enhancer activity were required to have *p* ≤ 0.05 for both tests. For anatomies where we called enhancer activity, an average of 64% of embryos showed activity. For the 71 cases where our rule indicated significant CCE activity in an anatomy, these anatomies displayed an average of 4× higher expression than the control plasmid, ranging from 1.6× at the lowest to 14×at the highest (Table 1 and Data File S2). No pair of CCEs displayed the same set of active anatomies, indicating that the observed enhancer activities were controlled by individual CCE effects rather than systematic biases.

**Table 1:** Examples of Expression Data.

PLASMID NAME	Control	CCE-rab11fip4a	X	CCE-abca1a	X	CCE-odz3	X	CCE-rfx2	X
**Total Embryos Scored By Voice-Recognition**	161	53		43		39		48	
**Raw Expression proportion**									
Forebrain:	0.3354	0.4340		0.3488		0.8205	2.4	0.3750	
Midbrain/Hindbrain:	0.2546	0.1509		0.3953		0.0256		0.1250	
Eye:	0.1800	0.2264		0.4419	2.5	0.0769		0.1667	
Ear/AboveHeart:	0.0680	0.0189		0.0698		0.2821	4.1	0.0208	
Heart:	0.3160	0.2264		0.0000		0.1538		0.1042	
Notochord:	0.1610	0.6038	3.8	0.3953	2.5	0.0769		0.1042	
Yolk/YolkExtension:	0.1550	0.2075		0.3953	2.6	0.1282		0.2708	
MidTrunk/AboveYolk:	0.0800	0.0943		0.0930		0.1538		0.0000	
Muscle:	0.1800	0.1509		0.3488		0.5128	2.8	0.6875	3.8
TailRegion:	0.1240	0.1887		0.1163		0.2051		0.1667	
**p-values prop.test**									
Forebrain:		0.1289		0.5000		5.48E–08		0.3693	
Midbrain/Hindbrain:		0.9147		0.0519		0.9983		0.9546	
Eye:		0.2941		0.0003		0.9089		0.5000	
Ear/AboveHeart:		0.8445		0.5000		0.0002		0.8127	
Heart:		0.8595		1.0000		0.9660		0.9969	
Notochord:		5.14E-10		0.0009		0.8627		0.7732	
Yolk/YolkExtension:		0.2512		0.0006		0.5694		0.0539	
MidTrunk/AboveYolk:		0.4904		0.5000		0.1373		0.9547	
Muscle:		0.6094		0.0146		1.84E-05		2.44E-11	
TailRegion:		0.1725		0.5000		0.1470		0.3028	
**p-values average 3 runs of wilcox.test**									
Forebrain:		0.1964		0.5680		0.0053		0.5038	
Midbrain/Hindbrain:		0.9497		0.1371		0.9961		0.9711	
Eye:		0.2282		0.0460		0.9572		0.7784	
Ear/AboveHeart:		0.9744		0.7036		0.0287		0.9525	
Heart:		0.9047		0.9981		0.9758		0.9950	
Notochord:		0.0058		0.0057		0.9847		0.8387	
Yolk/YolkExtension:		0.2801		0.0276		0.7832		0.1060	
MidTrunk/AboveYolk:		0.5277		0.5800		0.2014		0.9966	
Muscle:		0.7513		0.0524		0.0205		0.0051	
TailRegion:		0.4191		0.5419		0.1681		0.2205	

Raw Expression Proportion (top) displays the fraction of surviving embryos with expression in each anatomy. X column shows the ratio of expression fraction for statistically significant anatomies. We consider anatomies as significant if they have p≤0.05 for both the Wilcoxon and Proportions test (middle and bottom). The full dataset can be found in Supplemental Data File S2.

**Figure 2:**
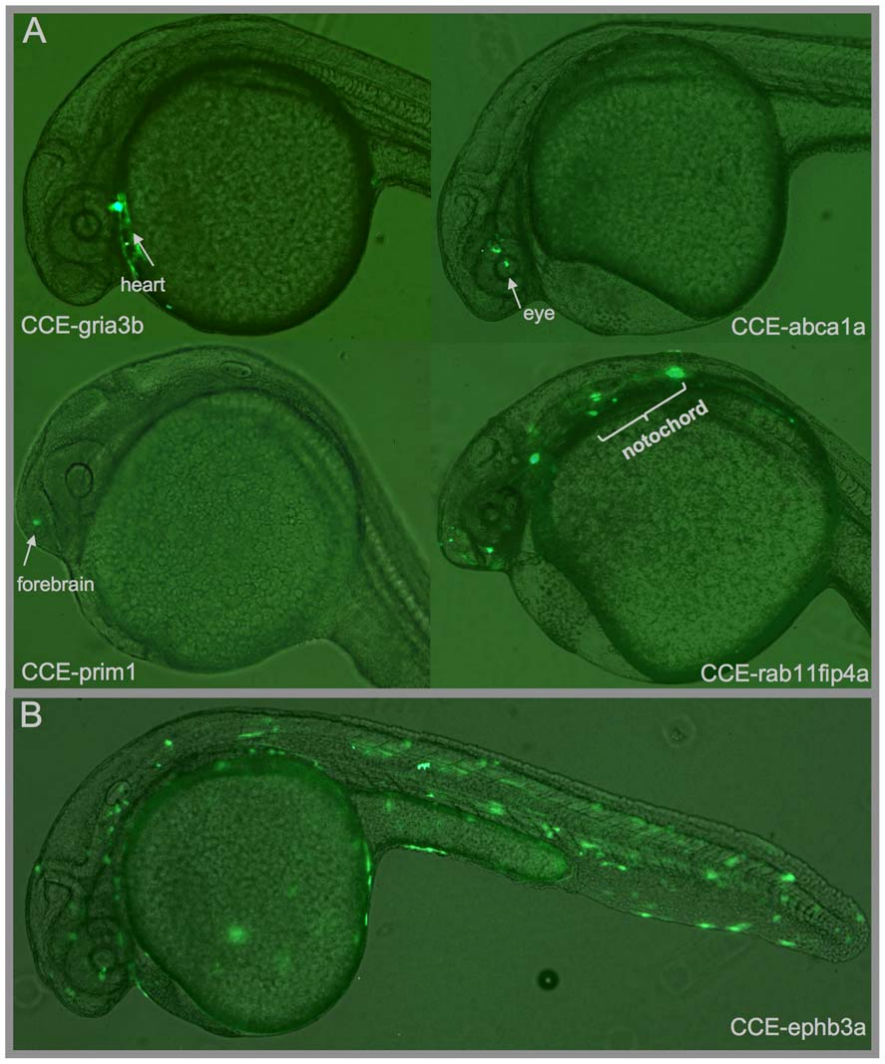
Specific and Non-Specific CCE Activity. (A) Examples of Specific CCE Activity. CCEs from the genes gria3b, rab11fip4a, prim1, and abca1a each drove robust expression in a finely localized anatomical region. Overall, 14 CCEs produced this type of specific expression (defined as expression in 4 or fewer anatomical regions). (B) This behavior contrasts with CCEs that drove robust but non-specific expression, such as CCE-ephb3a. 6 of the active CCEs drove nonspecific expression.

We observed that 24/31 CCEs (77%) drove clear GFP expression above the control. Although there was a small amount of mosaicism, 20/24 CCEs drove expression in at least one anatomy at a level significantly greater than the control (Figures S9, S10 and S11). Each of these CCEs drove expression in >35% of embryos, including 4 with expression in >80% of embryos (Table S1). 14/24 (58%) CCEs were anatomy-specific, defined as having activity in 4 or fewer anatomies. Examples of anatomy-specific CCEs, in contrast to the non-specific activity of CCE-ephb3a, are shown in Figure 2. The 7 CCEs which we assessed as non-functional exhibited expression in<5% of embryos. The fraction of CCEs which we observed to have enhancer activity was much higher than that of random sequences with cryptic activity, as measured by Sanges et al. in a transient co-injection assay (17%) [32].

**Figure 3:**
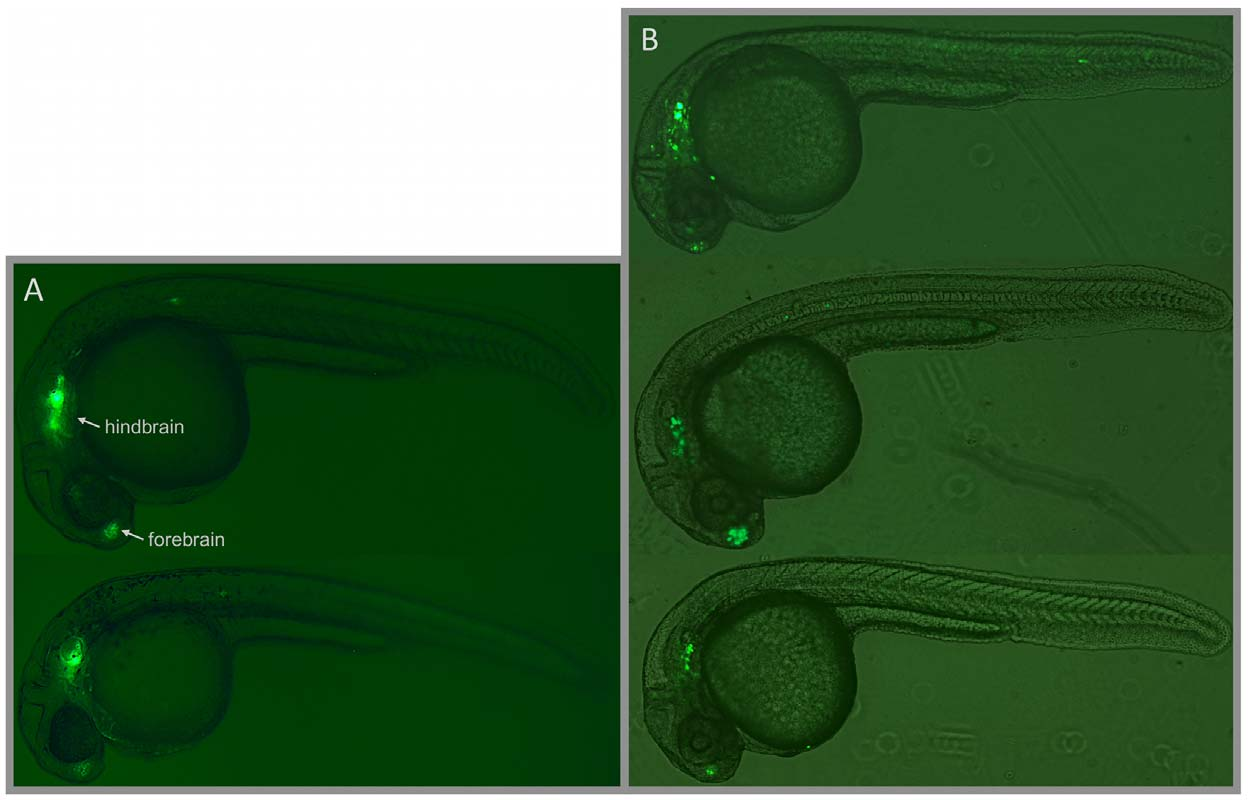
Comparison of CCE-lmo1 Stable and Transient Transgenic Expression. (A) Stable transgenic F1 embryos from two independently generated lines displaying strong forebrain and hindbrain expression. Supplementary Figure S3 shows this behavior in a larger group of stable transgenic embryos. (B) Similarly, three transient transgenic embryos injected with CCE-lmo1 display analogous forebrain and hindbrain expression.

To further confirm the validity of our assays, we made transgenic lines for one CCE: CCE-lmo1, which we chose because of its strong expression in the transient assay (see Methods). Two transgenic lines displayed very strong GFP expression in the forebrain and hindbrain, with excellent correspondence to the transient CCE-lmo1 expression. A comparison of stable vs. transient behavior is shown in Figure 3 (and Figure S3). The other two lines also showed the same pattern, but with a weaker background likely due to positional effects. This result is consistent with previous findings that activity measured in Tol2-based transient enhancer assays can be recapitulated in stable transgenics [3], [26], [33].

### Coding and Noncoding Enhancers are Similar in Both Activity and Tissue-Specificity

In a previous study we reported that 76/101 of CNEs, chosen by criteria similar to those used for the CCEs, exhibited enhancer activity as measured using Methods similar to those applied to CCEs [3]. We have since performed additional experiments to raise these numbers to 105/147 (∼71%). Our observed CNE enhancer rate is comparable to that found in other studies that have tested CNEs under conservation criteria relatively similarly to ours [34], supporting our experimental approach.

**Figure 4:**
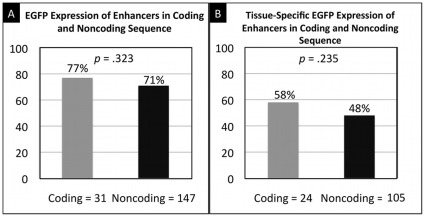
CCE and CNE Activity and Tissue Specificity. (A) Comparison of the fraction of enhancers active in conserved coding elements (CCEs) and conserved non-coding elements (CNEs). CCEs and CNEs exhibit similar enhancer activity levels, with no significant difference in activity. (B) Comparison of the fraction of enhancers exhibiting tissue specificity in CCEs and CNEs. While CNEs are marginally less tissue-specific, the difference is not statistically significant.

As shown in Figure 4A, enhancer activity rates are not significantly different between CCEs and CNEs (coding = 24/31 vs. noncoding = 105/147, *p* = .323). This indicates that there is no significant bias of enhancer function for conserved coding versus conserved noncoding DNA in zebrafish. Consistent with this locational independence, active and inactive CCEs show no difference in their average location within genes (Wilcoxon test on exon-rank, *p* = .49). Furthermore, we compared tissue-specificity (≤4 anatomies) for the active CCEs and CNEs. As shown in Figure 4B, 14/24 (∼58%) coding enhancers are tissue specific, and 50/105 (∼48%) noncoding enhancers are tissue specific, with no significant difference between the two classes (*p* = .235).

We have previously shown that for CNEs with greater than 60% human-zebrafish conservation, increased conservation cannot distinguish active and inactive CNEs [3]. Analogously, increased conservation did not associate with greater propensity for enhancer activity in our CCE set. 11 CCEs were the most conserved exons in their containing genes, yet only 54% of these yielded expression in at least one anatomy, lower than the overall activity rate (see Data File S1). For CCEs with conservation of 70–79%, 6/9 CCEs drove significant reporter expression; with conservation of 80–89%, 12/18 CCEs drove expression. Thus there was no increase in detecting enhancer activity with conservation. Similarly, 4-fold site conservation was not stronger for the sequences with activity (data not shown). The CCEs we tested span a range of AT (34%–61%) and GC (38%–65%) contents. The five most AT-rich CCEs all drive GFP expression, as do the five most GC-rich CCEs. We found no significant difference in activity for AT-rich CCEs. For example, for sequences with AT content >50%, 12/14 CCEs show activity, while for sequences with AT content<50%, 12/17 CCEs show activity. These ratios are not statistically different (*p* = 0.57). This independence of activity from GC content is similar to what has previously been observed for CNEs [3].

### Activity Patterns of CCEs

**Figure 5:**
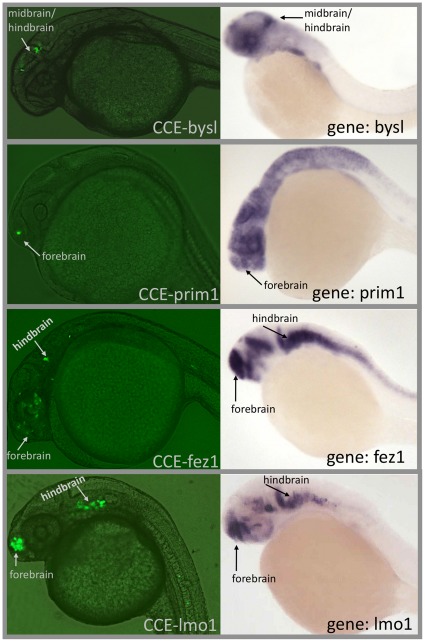
Representative images of CCE expression and host gene expression (mRNA *in situ* hybridization data from ZFIN) for 4 CCEs, showing overlap between CCE activity and host gene expression.

To investigate the target genes of CCE enhancers, we compared CCE activity to the anatomical expression of the gene in which the CCE resides (hereafter termed “host” gene) using ZFIN anatomy tags (see Methods and Figure S4). Our dataset contains 20 active CCEs for which *in situ* mRNA expression of the zebrafish host gene is available [35]. 12/20 CCEs display activity overlapping at least one anatomy of host gene expression in the 22–30 hours post-fertilization (hpf) time period (Data File S3). This is much more overlap than when CCE activity is compared to the expression of 100 sets of 20 random genes (3.6±1.8, see Methods). Furthermore, the CCE enhancer activities are more similar to expression of the host gene than that of neighboring genes. Significantly fewer CCEs have activity overlapping the expression of either the upstream or downstream gene (upstream 4/20: *p* = 0.02 and downstream 2/20: *p* = 0.003). This suggests that the target of a coding enhancer is often the gene in which the enhancer resides. Figure 5 displays examples of CCE activity consistent with expression of the host gene. For example, CCE-lmo1 is a conserved exon of a transcriptional regulatory gene, lmo1 [36]. CCE-lmo1 exhibits activity consistent with host gene expression in the forebrain and hindbrain of the nervous system (see also, Figure S5). Likewise, fasciculation and elongation protein zeta-1 (fez1) is active in anterior nervous system development [37], and CCE-fez1 displays expression in these areas. Similar behaviors are observed for CCE-bysl and CCE-prim1.

In some of the non-overlapping cases, there is evidence for host gene expression in the anatomies where the CCE is active. For example, CCE-gria3b and CCE-islet1 both display strong heart expression (see CCE-gria3b: Figure 2A, CCE-islet1: Figure S6). While ZFIN lacks mRNA *in situ* heart annotations for either, glutamate receptor *gria3* has been reported active in mammalian heart tissue [38], and *islet1* has been reported in both mammalian [39] and zebrafish heart [40]. Further investigation of such CCEs may be useful, both for clarifying the regulatory target and for their practical use in driving expression in the zebrafish heart [41].

**Figure 6:**
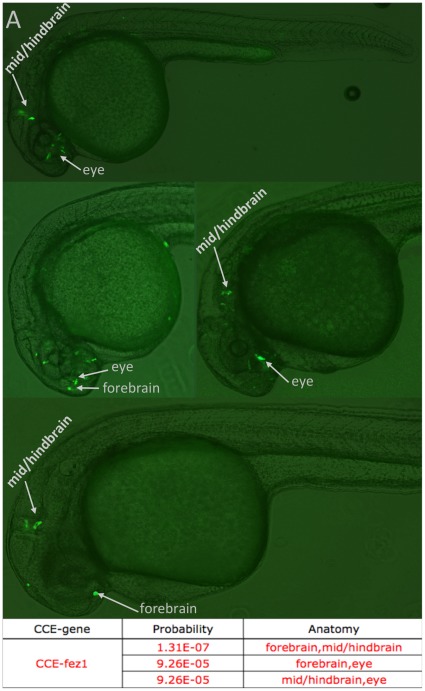
CCE-fez1 drives expression in multiple anatomies, with significant concurrent activity in forebrain, mid/hindbrain and eye. 4 representative embryos are shown.

A separate aspect of enhancer activity is that enhancers can be active in multiple tissues. Such concurrent activity may be functionally important, but mosaicism can often obscure recognition. An advantage of our method is that it yields activity annotations for all anatomies on an embryo-by-embryo basis, allowing us to quantitatively distinguish concurrent multi-anatomy activity from mosaicism (see Methods, Figure S7). Ten CCEs exhibit significant concurrent activity in at least one pair of anatomies (*p*-value<.05), as shown in Table S2. For example CCE-fez1 has significant concurrent activity in forebrain and midbrain (*p* = 1.3E-7), forebrain and eye (*p* = 9.2E-5), and midbrain and eye (*p* = 9.2E-5) (Figure 6).

### CCEs are Targeted by Enhancer-Related Histone Modifications

Histone3 Lysine4 monomethylation (H3K4me1) has previously been associated with ∼30–40% of enhancers in mammalian non-coding regions [42]–[47]. Aday et al. previously tested 6 sequences with H3K4me1 marks in zebrafish and showed that 4 exhibited enhancer activity [48]. We analyzed the prevalence of this mark in zebrafish at the same developmental timepoint as our enhancer experiments, using an H3K4me1 ChIP-seq dataset specific for zebrafish whole-embryo at 24 hours [48]. In our dataset, 9/24 (37.5%±9.8% s.e.m.) of our CCEs with enhancer activity have an H3K4me1 mark at that timepoint. In contrast, a much smaller fraction of other exons in those genes show this mark (37/358, 10.3%±1.6%). Similarly, H3K4me1 prevalence in CCE enhancers is much higher than the fraction of exons genome-wide which show the H3K4me1 mark (8874/110461, 8.0%±0.08%). In our prior dataset of validated CNE enhancers from zebrafish, 34/81 (42%±5.4%) have an H3K4me1 mark. This prevalence is not significantly different from CCE enhancers (*p* = .56). The H3K4me1 mark is more common in exons of developmental genes (942/6741, 13.9%±0.42%) than in exons genome-wide. On a gene-wise level, 517/813 (63.6%±1.7%) of developmental genes have at least 1 exon with H3K4me1 binding, in comparison to 6362/13588 (46.8%±0.43%) of genes overall. This is also higher than the prevalence of H3K4me1 in size-controlled intronic regions. Only 34.7%±0.42% of such intronic regions contain an H3K4me1 site. These findings indicate that H3K4me1 is active in the coding sequences of developmental genes, and that H3K4me1 has a similar functional importance for enhancers in noncoding and coding regions.

H3K4me1 is not a perfect predictor of enhancer activity, since only a subset of active CCEs show the mark. We note that in the more comprehensively characterized human ENCODE datasets, recent algorithms to predict enhancer activity from histone modifications (including H3K4me1) also have false negative rates of 20–40% [43]. We also considered whether these active CCEs might function as promoters rather than enhancers in their native context, as H3K4me1 can also occur at promoters. If this were the case, we would expect to find transcripts often beginning adjacent to CCE sequences. Using the UCSC EST database, we did not find an increase in adjacent transcripts. CCEs that drove significant anatomical activity were not more likely than those without activity to have ESTs that begin adjacently (15/20 = 71% vs. 5/7 = 75%, respectively).

### Coding Exons are Commonly Bound by Enhancer-Related and Histone Modifying Transcription Factors

p300 is a bromo-domain histone acetyl-transferase protein that has been associated with enhancers found in noncoding regions. To further determine whether enhancers are likely to be common in coding regions, we reanalyzed the mouse p300 ChIP-seq data of Visel et al. using CCDS coding exons, a stringently annotated set of conserved exons between mouse and human [14], [49]. 172/5118 (3.3%±0.24%) of p300 binding sites overlap a CCDS coding exon, which is higher than the fraction of the mouse genome covered by CCDS exons (1.0%).

In addition, we analyzed the clustered ChIP-seq human transcription factor dataset from ENCODE [50] and determined overlap with human exons. 24095/326254 (7.3%±0.04%) coding exons are bound by at least one transcription factor. The top 5 exon-bound transcription factors are HEY1 [51], TAF1 [52], BAF155 [53], POU2F2 [54] and c-MYC [55], all of which have been shown to be associated with enhancer activity. These factors are involved in both sequence-specific binding (Hey1 and c-MYC bind the E-box [56], [57]) and histone modifications (TAF1, BAF155, POU2F2, c-MYC [57]–[60]). This provides further support for transcription regulation and enhancer activity in coding sequence.

### CCEs May Contain Multiple Overlapping Functions

**Figure 7:**
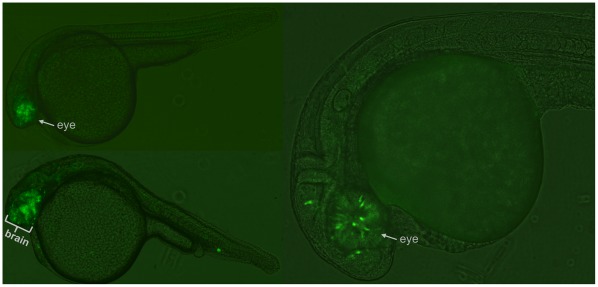
CCE-sfrs3b, an alternatively spliced exon, is shown here to drive enhancer expression in the eye and brain, despite poison cassette activity of the exon.

An alternate hypothesis for the function of highly conserved coding sequences has been proposed to be “poison cassettes” [61]. According to this hypothesis, certain exons may be spliced-in as alternative exons, and these poison-cassette exons then invoke mRNA degradation through the nonsense mediated decay (NMD) pathway via a premature stop codon. Lareau et al. previously showed that poison cassette exons are also highly conserved, suggesting that the high conservation in such exons may be related to poison cassette activity. We were curious whether a poison cassette exon would also display enhancer activity, as this would suggest that the strong conservation of poison cassette exons is coincidental to their NMD activity. We tested the enhancer activity of CCE-sfrs3b, an ortholog to the mammalian poison cassette exon in the splicing-regulator SRFS3 (Srp20) studied in Lareau et al. This CCE has an internal stop codon and overlaps 23 known ESTs. Although the human ortholog of this exon exhibits NMD-related poison cassette behavior, in zebrafish CCE-sfrs3b is a robust enhancer and displays concurrent activity in the brain and eye (Figure 7). In addition, CCE-sfrs3b has strong human-zebrafish sequence conservation both before and after the stop codon (each 83% id). This conservation pattern is consistent with homogeneous selection on a larger enhancer element, in addition to any constraints related to poison cassette activity or the amino acid sequence.

## Discussion

We have shown that conserved coding sequences often act as enhancers, with activity, tissue-specificity and protein-binding characteristics similar to highly conserved noncoding sequences selected by analogous criteria. While we tested only 31 sequences, 168 sequences met our screening criteria of human-teleost conservation and overlap with genes active during forebrain development. At our observed success rate (77%), this would imply ∼129 coding enhancers in the zebrafish genome. In all likelihood this is an underestimate, as there may be many coding enhancers that do not meet our selection criteria. In any case, our work demonstrates that even sophisticated regulatory functions such as enhancers may occur commonly in protein coding sequence.

These experiments clearly verify the coding enhancer hypothesis [62], [63]. Previously, Dong et al. computationally investigated whether exonic remnants of duplicated zebrafish coding sequences might contain enhancers, finding that synteny, conservation, and epigenetic data (from mammals) supported some exons having enhancer activity. However, they experimentally tested only one exonic remnant, a noncoding sequence originating from a duplicated zebrafish Elp4 exon (∼200 bp). They observed that the exonic remnant influenced enhancer activity when contained in a larger piece of noncoding sequence (∼2000 bp), but the exonic remnant alone was unable to drive consistent expression. A similar issue confounds the interpretation of a study by Lampe et al [21]. They showed that a 1.25 kb sequence containing the first exon, intron and partial second exon of Hoxa2 had some developmental enhancer activity. However, the first exon was not shown to have developmental activity alone. Our experiments provide a more direct demonstration that coding regions contain enhancer functions, as almost all CCEs correspond exactly to a single coding exon. In support of our findings, during the processing of this manuscript another group communicated to us evidence for transcriptional enhancers in coding exons that regulate nearby genes [64].

### Protein vs. Enhancer Function

The observation of prevalent coding enhancers is counterintuitive given that protein-coding constraints would be expected to conflict with other functions in the same location. However the degree of conflict depends on the amount of evolutionary constraint associated with both the protein and other function. Consider first the constraint associated with protein function. Previous studies have shown that 70% of amino acids in a protein can be altered while maintaining structure and function [65], [66]. This indicates that if an enhancer were to arise in a coding region, there would be substantial flexibility in the amino acid sequence to accommodate the enhancer function, in addition to the flexibility of changing synonymous sites. Consistent with this idea, we have previously shown that for 6-mer motifs, nucleotide-level pressures have commonly superseded protein-level constraints [10]. This malleability of protein sequences suggests that coding enhancers need not have much higher conservation than other coding sequences.

The level of constraint associated with enhancer activity remains controversial, as enhancers vary widely in their sequence conservation. Conservation-blind enhancer identification approaches in noncoding regions have suggested that enhancers are typically under strong sequence constraint. McGaughey et al. tiled intergenic regions around the phox2b locus in zebrafish and found that ∼40% of sequences with enhancer activity had ≥75% zebrafish-human conservation in a block ≥100 bp [34]. Kim et al found that noncoding enhancers identified by p300 and methylation marks had phastCONS conservation scores (peak ∼0.4) higher than the background (∼0.1) [46]. Visel et al. found that ∼90% of p300 identified enhancers are under evolutionary constraint [14]. However, there are also substantial deviations from this typical behavior. Some sequences without overt conservation act as enhancers [26], [34], [67]. Also, it has been found that 40% of ultraconserved sequences display no enhancer activity, though this conclusion is limited by the small number of experimental conditions that have been probed [16]. These variations make it difficult to determine how many bases are necessary for each enhancer. Still, the lengths of highly conserved coding and noncoding sequence blocks suggest that enhancers span many dozens of bases [68].

### Predicting Coding Enhancers

How should enhancers in coding regions be predicted? Given that enhancer-protein conflict appears to be weak, this question is essentially the same as for predicting enhancers in noncoding regions. In other words, occurrence in developmental loci and relatively high conservation (e.g. our criteria of >60% fish-human ID over 100 bp) are important features, the application of which should yield true positive rates of ∼3/4. These criteria tend to overlap – for the genome-wide set of exons with 1∶1 orthology, there are 8,693 exons (avg. conservation 77%) and many are from developmental genes. There are 6274 exons from developmentally expressed zebrafish genes, and 61% are at least 60% similar to human. Even nonconserved sequences in developmental loci may have substantial rates of enhancers. For example, McGaughey et al found enhancer activity in 4/13 blocks of noncoding sequence near the zebrafish phox2b developmental gene lacking conservation to fugu, tetraodon, human or mouse [34].

Extensions of sequence-based prediction approaches, e.g. through superior neutral background models [69], may yield improvements in true positive rates. However, we do see that conservation and likelihood of enhancer activity have little correlation at the high end of the conservation spectrum, and predictive approaches based on presence of transcription factor binding site sequence motifs, while beneficial, still have substantial error rates [2], [31], [70]. Given these complexities, it will be difficult to elucidate predictive features by testing “random” control sequences in embryos since the possible set of predictive features can not be adequately surveyed in any small control set. Recent approaches based on large-scale ChIP data for condition-specific epigenetic features [14], [71] are likely to be important if very high true positive rates are desired. Interestingly, the lack of association between extreme conservation and enhancer activity suggests that the most conserved sequences may be conserved because they have additional selective pressures, such as poison cassette activity, layered on top of the enhancer activity. Other pressures might include the exon-sharing that has been observed for Hox loci, binding sites for microRNAs, or effects related to mRNA structure [10], [72], [73]. These issues remain open, and it is likely that a much larger number of exonic sequences will have to be experimentally profiled and analyzed to resolve the interplay of such pressures.

Our work suggests that enhancers in coding regions target their own gene. This finding is consistent with the genomic regulatory block concept of Kikuta et al. that enhancers and their targets should remain syntenic through evolution [74]; CCEs are a more extreme form of this idea since the enhancer and target coincide. In addition to being advantageous for modularity, this behavior might be related to the mechanism of enhancer activity. For example, Kim et al. reported that transcription commonly occurs at enhancers [46] and Orom et al. found noncoding RNAs whose DNA sequences have enhancer activity mediated by the transcribed ncRNA [75]. Localization in transcribed regions would provide CCEs an inherent feedback system for regulation of the host gene.

Finally, this work sheds light on the many protein-binding, histone modification, and RNA-binding events in coding DNA which have typically been regarded as ‘experimental noise.’ Given that coding sequence can contain enhancer functions, it is likely that many of these events are functional as well. A number of recent disease studies have shown the functional importance of synonymous SNPs [76]–[78], and it is likely that similar functional events in coding sequences have been substantially under characterized.

## Materials and Methods

Additional CCE images and the program to calculate significant anatomies are available at the public website and database: http://bioinformatics.bc.edu/chuanglab/CodingEnhancer.

### Conserved Exonic Sequences and Ultra Conserved Regions

To determine a set of CCEs, we identified exons with mutual best BLAST hits among Ensembl RefSeq exons from zebrafish (dr6) and human (GrCH37) with E-value<1e^−10^ [79]. The average conservation of this set was 76.5% (8693 seqs, σ = 11.6%). Exons from the set of 8693 sequences were filtered for >60% conservation between zebrafish-human, >100 bp, and lacking *XhoI* or *BglII* cut sites. Because the expression assay is performed during development, a set of 250 human-zebrafish orthologous forebrain/developmental genes from Ensembl was used to filter potential experimental exons. 26 coding exons with unique primers were chosen from the 168 sequences that met these criteria based on considerations of exon rank and conservation level. MultiZ vertebrate 6-way CDS Fasta alignments from the UCSC Genome Browser were used for sequence analysis [80]. Cross-species sequence identity was calculated from positions wherein neither species exhibited a gap.

Additionally, 5 ultraconserved regions (UCRs) were chosen from Bejerano et al. [1]. 4 of these overlap known Refseq coding exons while the last one (CCE49 from SFRS3) overlaps an exon known from human and zebrafish EST data and Ensembl alternative transcript data. These UCRs also have >60% conservation and are from the forebrain/developmental gene list. UCR sequences were specified by liftover of the original hg16 coordinates to the hg19 and dr6 genomes. Alignments were based on available pairwise hg19-dr5 alignments in Galaxy [81], and correspondences with the dr6 sequences were verified manually.

### Plasmid Creation and Sequencing

Primers were designed using the Primer3 executable [82]. The primer search space was from the 25 bp within the CCE to 50 bp outside with a preference for sites exactly matching the end of the CCE. Genomic DNA was amplified from SH (Scientific Hatcheries) wildtype zebrafish using primer sequences with *XhoI* and *BglII* end cut sites.

The plasmid (pT2KXIGQ) is a modification of the Tol2 plasmid pT2KXIG [83]: the longer Tol2 arm was shortened by digestion with BglII and NruI, T4 fill-in and self ligation, and fragment XhoI-SalI (EF1a) was replaced with the E1B minimal promoter. Tol2 plasmids have been used by many groups for enhancer studies in zebrafish due to their decreased mosaicism and robust integration [7], [26], [48], [84]–[86]. Our construct was previously used in studies to characterize the presence/absence of enhancer activity for >100 CNEs, and to characterize fezf2 binding sites [3], [31], [87]. Inserts were ligated in upstream of an E1B minimal basal promoter 5′ to EGFP. The insert and reporter gene in the PT2KXIGQ construct are surrounded by ∼300 bp Tol2 sequences on each side, which improves the function of expression and integration of this vector compared to the full-length Tol2 plasmid [86]. The control plasmid was created by excision of the plasmid insert, isolation of plasmid backbone, removal of overhanging ends and T4 blunt-end ligation. All plasmid inserts were sequenced for quality verification, and 25/31 sequences were exact matches to the reference genome (Tübingen Wildtype). 5 differed by 1 base (CCEs bysl, ddx18, prim1, hif1an, erm, rab11fip4a) and 1 differed by 2 bases (sfrs3b).

### Zebrafish Embryo Injection

Pooled zebrafish embryos from AB and SH strains were collected within 10 minutes of fertilization. 150–300 embryos were injected per CCE with typically ∼130 surviving. The amount of injected plasmid DNA was consistent across CCEs and was very close to that in prior zebrafish enhancer studies [88]. The concentration of plasmid DNA aliquot was measured prior to injection on a Nanodrop spectrophotometer. CCEs were generally injected using 25 ng/µL plasmid and 30 ng/µL transposase (2 nL injection). However, if a CCE had no expression at 25 ng/uL, the concentration of plasmid and transposase was increased up to a maximum of 35 ng/µL and 30 ng/µL transposase. Likewise, if many embryos displayed abnormal development at the initial concentrations, the experiment was discarded and the concentrations of CCE and transposase were lowered to achieve normal development. For 29/31 CCEs, 2 separate sets of injections were performed on different days with different pooled WT fish crosses. The control plasmid was injected in 4 separate rounds of WT fish crosses.

### Zebrafish Transgenic Line

150 zebrafish embryos were injected with plasmid LMO1. At 24 hpf, 50 fish were chosen with strong and specific expression. ∼20 adults survived to adulthood and 6 were chosen to cross with wildtype zebrafish. 4 of the 6 crosses resulted in GFP expression, with around 30–40% of F1 offspring displaying GFP expression similar to transient LMO1 expression.

### Zebrafish Expression Scoring Using Voice Recognition

Embryos were visually scored for EGFP expression between 22–0 hours post-fertilization (judged by direct visualization of the 3-D living embryos from multiple viewing angles: dorsal, ventral, lateral, oblique, etc.) Representative white-light and fluorescence images were acquired at 5–20X. All CCEs and the control plasmid were tested in multiple independent runs. Subsets of embryos were anesthetized and plated (in sets of 15–20) onto inverted 96-well cell culture dish lids. Embryos were scored using the iPhone voice recognition application DragonDictation and a controlled-language anatomy for 10 anatomical sections as shown in Figure 1. A caveat is that our labeling does not distinguish all the formal anatomical regions found in ZFIN (e.g. not distinguishing anterior and posterior notochord). This is a compromise between the known zebrafish anatomies and the amount of feasible detail when many dozens of embryos are being observed. The mobile phone was placed by the microscope, freeing both hands for embryo sorting and scope operation.

Resulting text files were manually reviewed and processed by a PERL script and R Statistical Package [89], as shown in detail in Figure S2. We required the CCE to meet two significance criteria (*p*<.05) in order to be annotated with a particular anatomy. The first is a proportions test (prop.test) using all scored embryos. We required the fraction of all scored CCEs expressing in an anatomy to be significantly greater than that of the control plasmid. The second criterion accounts for variability over multiple rounds of injections as follows: the voice-data from all embryos in all injection sets for an individual CCE were shuffled and randomly partitioned into 5 groups. For each of the 5 groups, the fraction of embryos expressing was calculated. The values for these groups, as well as the values from shuffling and random partitioning of voice data for all injected sets of control embryos, were used as input for a rank sum test of means (Wilcox.test). We required the mean rank sum for the CCE to be significantly greater than the mean rank sum of the control (see Data File S2).

Of note, our criteria to classify a CCE as having significant expression in an anatomy are stringent. To determine enhancer activity, previous publications have used thresholds of ∼4% [5], ∼7% [29], and ∼10–20% [26] of embryos displaying GFP activity. The lowest activity rate that we called as a significant specific enhancer had >28% of embryos displaying expression (ear region), which showed 7% activity in the control. We also note that the (% expressing embryos) statistic exhibits a relatively bimodal distribution. Of the 31 CCEs tested, 10 of them have a (% expressing) value between 0 and 10%, which is the most common decile. The next most common decile is from 50–60%, with 9 CCEs in this range. Because of this bimodal behavior, classification of active CCEs is relatively insensitive to the threshold level.

To determine pairs of anatomies with concurrent activity, we assumed a null hypothesis of equal probability for the four cases: 00, 01, 10, and 11, where 0 and 1 indicate absence or presence of activity and the two digits correspond to the two anatomical regions. A co-regulation z-score was calculated as z = (N_11_–0.25* N_total_)/(N_total_ * 0.25 * 0.75)^1/2^ and a *p*-value was then calculated based on a Normal approximation (see Figure S7).

### Comparison of Enhancer Activity and Gene Expression

We downloaded the complete set of known anatomical annotations for every gene in the zebrafish genome from Zfin (10,746 unique genes). These annotations are based on literature-curated *in situ* hybridization and PCR data [35]. We then determined if the annotated expression domains of a given gene in the 22–30 hour period of development (stages Segmentation:26+ somites to Pharyngula:Prim15) overlapped any of the 10 anatomical regions in each CCE’s activity annotation using custom PERL scripts (see Figure S4). This approach removes subjectivity in manual comparison of images and also allows one to use the full set of ZFIN gene expression data, some of which do not have images available.

The set of possible ZFIN anatomies was created by text-matching anatomical descriptions for CCE significant anatomies to ZFIN anatomical IDs. We also allowed for matches to IDs one sub-level down in the ZFIN anatomical hierarchy to account for variations in the resolution of anatomical annotations. For example, “forebrain” = ZFA:0000109. The immediate sub-level down from forebrain contains the following terms: “diencephalon” = ZFA:0001343, “eminentia thalami” = ZFA:0007010, “forebrain ventricle” = ZFA:0000101, “telencephalon” = ZFA:0001259 and “telencephalon diencephalon boundary” = ZFA:0000079. These 6 IDs were also used to query the ZFIN gene expression database for matches to forebrain enhancer activity. This matching flexibility is important when the mRNA expression covers a diffuse area. For instance, CCE-ddx18 displays overlapping expression with ddx18 mRNA *in situ* hybridization at in the forebrain and midbrain at ∼30 hpf, but the expression of the eye and tectum make it difficult to determine whether there is agreement on a finer scale (Figure S8).

To compare CCE expression to random genes, the host/upstream/downstream and genes for the CCEs were removed from the Zfin wildtype expression file, as were miRNA genes. List::Util ‘Shuffle’ Perl module was used to randomly pick 20 genes and assign to CCEs as “host genes.” The number of anatomies shared between the CCE and the random host gene was then counted. This process was repeated 100 times. The mean, standard deviation and proportions analysis was done using the R Statistical Package.

### Comparison of CNEs and CCEs

To treat CNEs and CCEs equally, coordinates from experimentally tested CNEs in Li et al [3] were lifted to the Zv8 build using UCSC Genome Browser Lift Over [80]. Prop.test from R Statistical Package [89] was used to calculate significance.

### Histone Methylation

H3K4me1 binding sites were obtained from the recently published data of Aday et al. [48] and used for analysis. Zebrafish (Zv9) exons and introns were obtained from UCSC Genome Browser CDS Fasta records. The set of genes for the H3K4me1 analysis was determined by obtaining the flat database file “Expression Data for Wildtype Fish” from ZFIN. Developmentally expressed genes were obtained by filtering anatomical staging data for genes expressing in 0–30 h of fertilization by excluding stages beyond Prim15 in the ‘EndStage’ column. To ensure higher quality of expression, we kept records with RNA *in situ* hybridization probe quality > = 3. Exons were obtained by converting the Zfin gene ID to the RefSeq gene name, then using CDS Fasta records from the UCSC Genome Table Browser. BedTools was used for overlap of histone modification markers and zebrafish exons. Uncertainties listed in the main text refer to standard error of the mean of a binomial variable given the observed mean value and number of samples. For the EST analysis, zebrafish ESTs were downloaded from the UCSC Genome Browser. Using BedTools IntersectBed, 1 kb on either side of the CCE was used to count ESTs that intersected but did not completely overlap the 1 kb frame.

### Four Fold Site Conservation

Human (hg19) and zebrafish exons (Zv8) for tested CCEs and all exons from 100–1000 bp were extracted using CDS Fasta data from the UCSC Genome Browser. Sequences were searched for aligned 4-fold synonymous codons, and a minimum of five such codons were required for further analysis. Four-fold sites were extracted and the p-distance was calculated by counting the number of conserved sites divided by the total number of sites. Random exons were extracted using PERL to randomly shuffle the set of all exons. Random exons were required to have alignable coding sequence between human and zebrafish. The R Statistical package was used for the unpaired Wilcoxon rank sum analysis.

### p300 Analysis

p300 peaks were obtained from Visel et al. 2009 [14]. Peaks were intersected with unique mm9 CCDS exons obtained from the UCSC Genome Browser (154,896 exons) using IntersectBed from BedTools [49], [90]. Exons with duplicate or overlapping annotations and any exons<3 bp or >16 kbp were removed.

### Clustered Transcription Factor Binding Site Analysis

TFBS clusters on 8 human cell lines were obtained from the UCSC Genome Browser Encode Project [50]. Human exons from Hg18 were obtained from CDS Fasta records. The TFBS cluster score was > = 500 (maximum possible 1000), and we required 100% coverage of the exon by the TFBS cluster. BedTools was used for overlap of Hg18 exons and clustered TFBS.

## Supporting Information

Figure S1 Plasmid Design and Injection. Flanking Tol2 sequences integrate the control or experimental cassette into the zebrafish genome after injection with plasmid and transposase mRNA at the 1-cell stage.
(TIF)

Figure S2 Processing Voice-Operated Anatomical Expression Analysis. A schematic representation of how the proportions and Wilcoxon rank-sum test compare CCE-slc1a2 expression in the forebrain and yolk to the background expression of the control plasmid lacking an insert. Only anatomies with *p*<.05 by both tests were considered significant. The full datasheet containing p-values for both tests and proportions for experimental inserts and the control is Supplemental Data File S2.
(TIF)

Figure S3 A group of stable transgenic embryos (F1) derived from embryos injected with CCE-lmo1. Injected embryos were selected for forebrain and hindbrain expression and then crossed with wildtype zebrafish to yield the F1 generation.
(TIF)

Figure S4 Anatomy Comparison Using Zfin. For the host/upstream/downstream genes, the Zfin gene expression database was queried using anatomical terms corresponding to our CCE anatomies. The number of unique shared anatomies was counted for each CCE-gene comparison. CCEs with at least 1 shared anatomy with the gene were assigned a score of “1” while CCEs without were assigned “0.” The number of CCEs with a match was counted. Since there were 20 CCEs to be tested, in the randomized control the same procedure was used but with 100 random sets of 20 genes.
(TIF)

Figure S5 Comparison of CCE-lmo1 expression to Zfin stages. CCE-lmo1 maintains strong similarity to the mRNA *in situ* hybridization of LMO1 throughout a large window of development (22–42 hpf).
(TIF)

Figure S6 CCE-islet expression in the heart.
(TIF)

Figure S7 Concurrent Anatomical Activity Schematic. Each anatomy pair is compared to a null expectation of equal likelihood of expression in each of four cases: 00, 01, 10, 11. The first position represents the first anatomy, the second position represents the second anatomy. A 0 represents no expression and 1 represents expression.
(TIF)

Figure S8 CCE-ddx18 displays expression in the forebrain and midbrain, consistent with annotations in the ZFIN database. However, the diffuse expression patterns around the tectum and eye (particularly at ∼22–24 hpf) make it difficult to visually determine whether there is agreement on a finer scale.
(TIF)

Figure S9 Images from the 20 significant CCEs and their corresponding anatomies. Images are labeled with (CCE-GeneName, ExonNumber), and the significant anatomy for each CCE is labeled. To view more images for each CCE, please visit: http://bioinformatics.bc.edu/chuanglab/CodingEnhancer
(TIFF)

Figure S10 Images from the 20 significant CCEs and their corresponding anatomies. Images are labeled with (CCE-GeneName, ExonNumber), and the significant anatomy for each CCE is labeled. To view more images for each CCE, please visit: http://bioinformatics.bc.edu/chuanglab/CodingEnhancer
(TIFF)

Figure S11 Images from the 20 significant CCEs and their corresponding anatomies. Images are labeled with (CCE-GeneName, ExonNumber), and the significant anatomy for each CCE is labeled. Note that CCE-ddx5 has voice-expression data but lacks an image of yolk expression. To view more images for each CCE, please visit: http://bioinformatics.bc.edu/chuanglab/CodingEnhancer
(TIFF)

Table S1 Expression and Count Statistics for CCEs Evaluated for Whole Embryo (non-anatomy based)
(TIF)

Table S2 CCE concurrent activity. 10 CCEs display concurrent activity in at least two anatomies with *p*<.05 compared to a null expectation of equal likelihood of expression in each of four cases: 00, 01, 10, 11. The first position represents the first anatomy, the second position represents the second anatomy, 0 represents no expression and 1 represents expression. CCEs with *z*-score>3 are highlighted in red.
(TIF)

Data File S1 Data file of zebrafish-human exon conservation. ExonConservation_AllExonsInGene tab: The average of all exons in the gene is highlighted in yellow, the CCE tested in marked in red text. Exon-Cons,Total,Rank tab: a table of the average conservation, the CCE conservation, the exon rank of the CCE, and a count of exons in the gene.
(XLS)

Data File S2 Data file of wilcox.test and prop.test scores. The raw expression proportion (top) displays the fraction of surviving embryos with expression in each listed anatomy. X (green) shows the ratio of expression fraction for statistically significant anatomies, which are highlighted in yellow. We consider anatomies as significant if they have p≤0.05 (shown in red) for both the Wilcoxon and Proportions test (middle and bottom).
(XLS)

Data File S3 Data file of CCE activity patterns. CCE_HostGeneComparison tab: lists the CCE, the anatomies of experimental GFP expression, the ZFIN ID of the CCE-containing gene, the anatomies of gene expression in ZFIN database. Expression information for the closest upstream and downstream gene is also listed. RandomlyAssignedGenes tab: Counts of matching expression for 100 sets of 20 randomly assigned genes. CCE_GENE_UPSTREAM_DOWNSTREAM tab: ZFINID and common gene name for the CCE-containing gene, and the closest upstream and downstream genes. ExtendedAnatomy tab: Relational anatomy tags from the ZFIN database assigned to the anatomy tags used to visually score GFP in zebrafish embryos.
(XLS)

Data File S4 Expression, location and conservation of 31 Conserved Coding Elements. 20 CCEs display significant expression: 14 CCEs display significant specific expression (≤4 anatomies) and 6 display significant non-specific expression. In addition 4 CCEs display weak expression, and 7 CCEs fail to display expression. Sequences with Ultra-Conserved Regions are marked as (UCR).
(XLSX)
