# Supplementary figures and images for: Transcriptional Enhancers in Protein-Coding Exons of Vertebrate Developmental Genes
Source: PLoS One. 2012 May 2;7(5):e35202. doi: 10.1371/journal.pone.0035202 (PMC3342275; doi:10.1371/journal.pone.0035202)

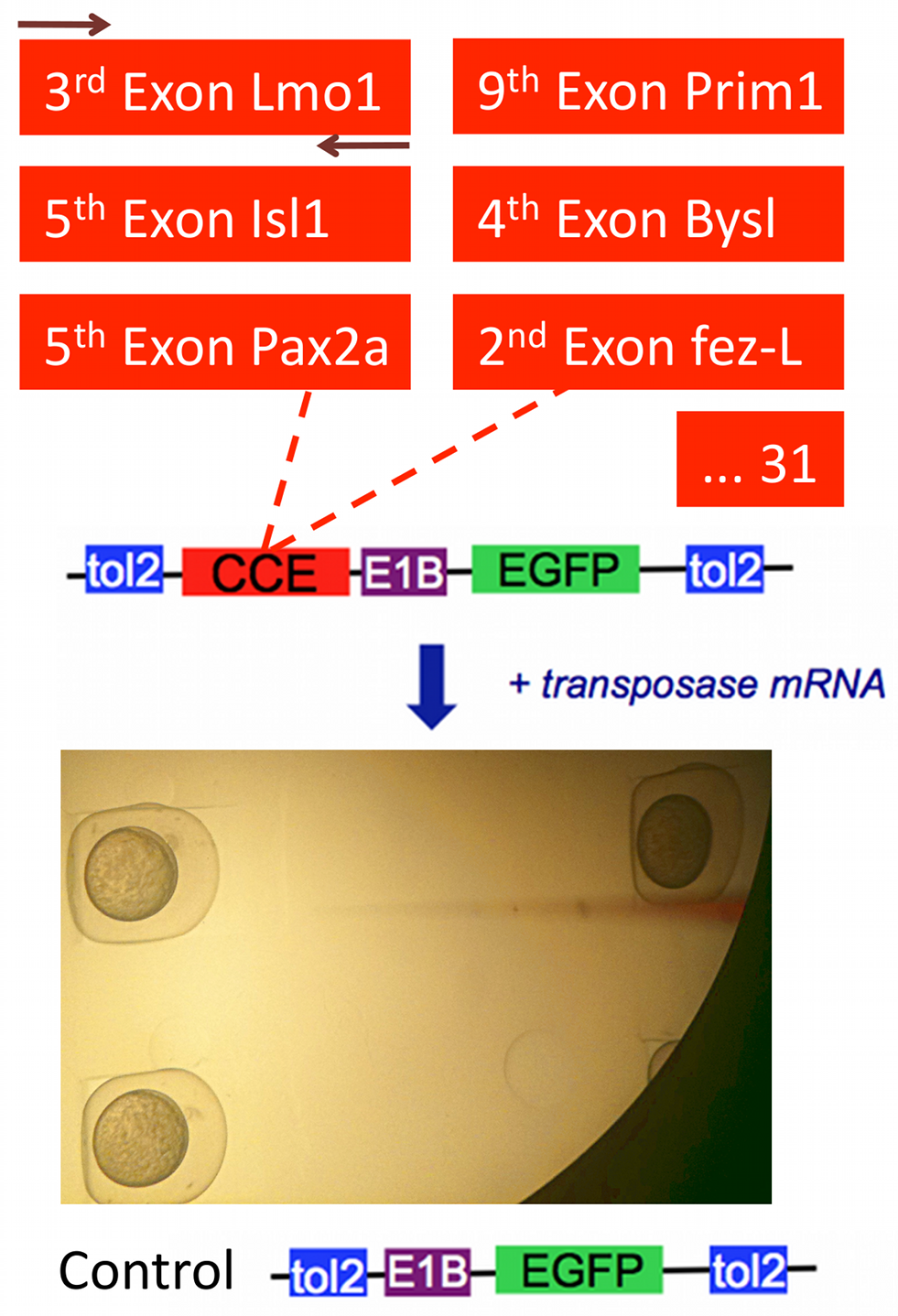

Supplement: Figure S1 — Plasmid Design and Injection. Flanking Tol2 sequences integrate the control or experimental cassette into the zebrafish genome after injection with plasmid and transposase mRNA at the 1-cell stage. (TIF) [file pone.0035202.s001.tif]

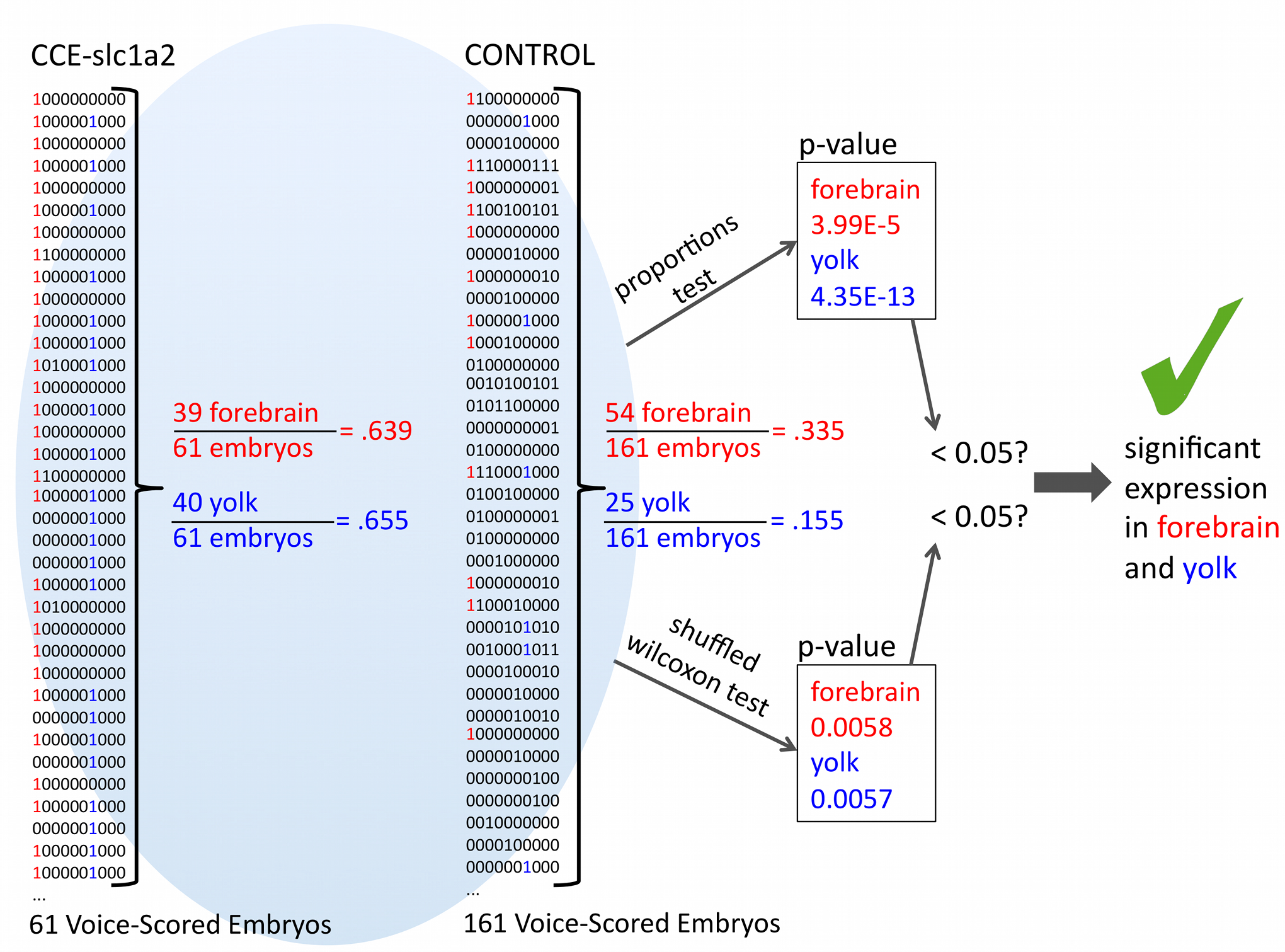

Supplement: Figure S2 — Processing Voice-Operated Anatomical Expression Analysis. A schematic representation of how the proportions and Wilcoxon rank-sum test compare CCE-slc1a2 expression in the forebrain and yolk to the background expression of the control plasmid lacking an insert. Only anatomies with p<.05 by both tests were considered significant. The full datasheet containing p-values for both tests and proportions for experimental inserts and the control is Supplemental Data File S2. (TIF) [file pone.0035202.s002.tif]

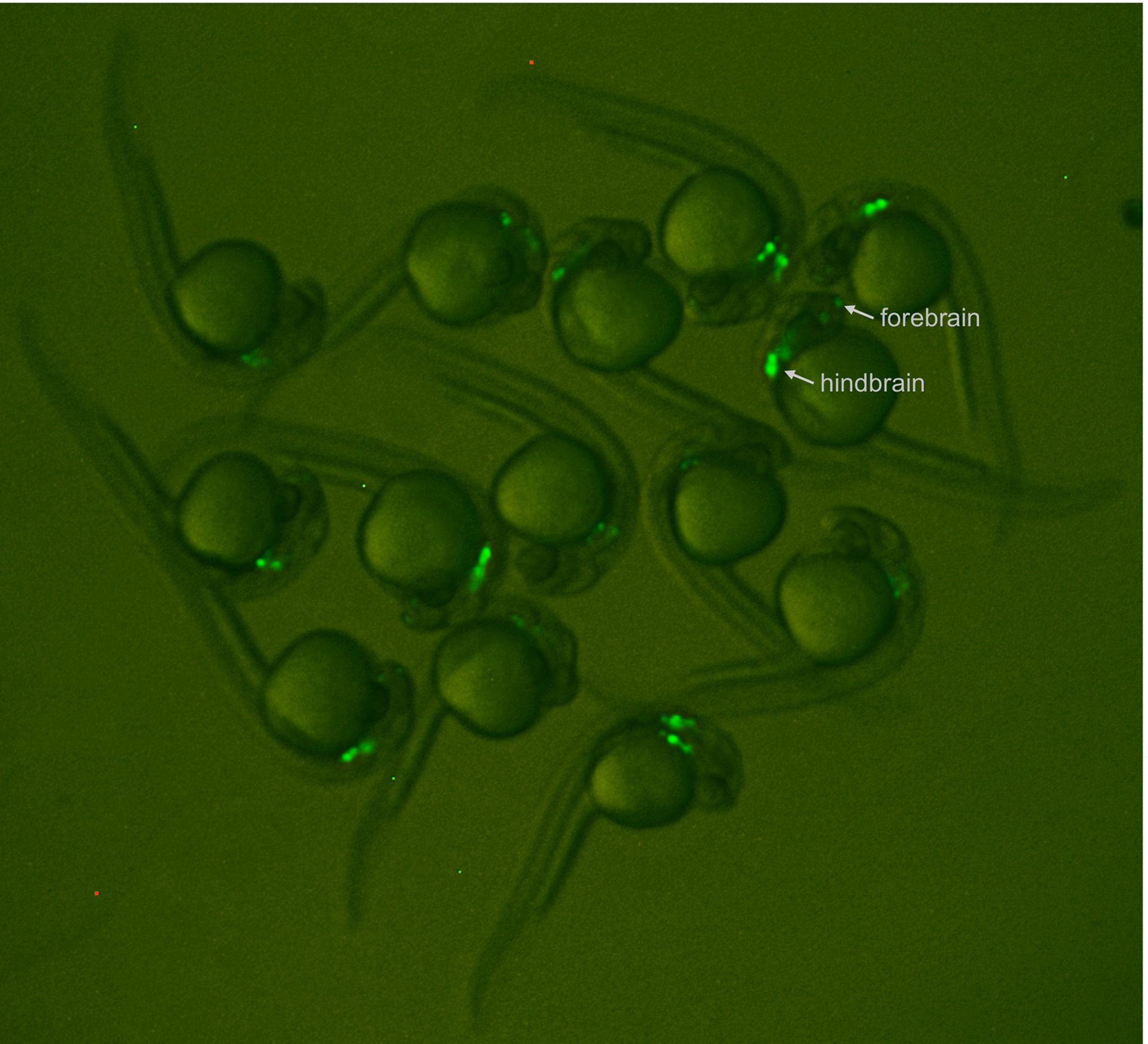

Supplement: Figure S3 — A group of stable transgenic embryos (F1) derived from embryos injected with CCE-lmo1. Injected embryos were selected for forebrain and hindbrain expression and then crossed with wildtype zebrafish to yield the F1 generation. (TIF) [file pone.0035202.s003.tif]

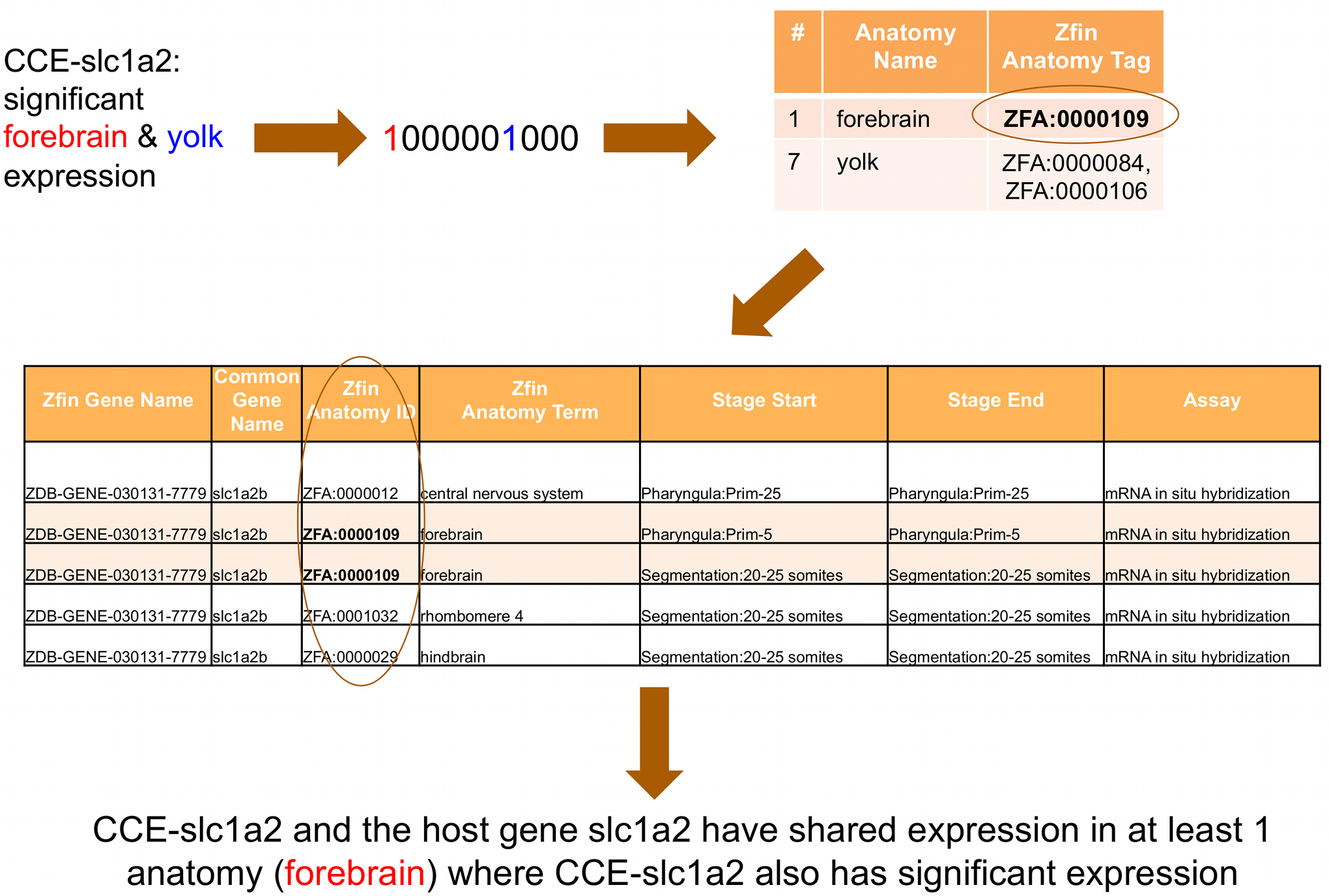

Supplement: Figure S4 — Anatomy Comparison Using Zfin. For the host/upstream/downstream genes, the Zfin gene expression database was queried using anatomical terms corresponding to our CCE anatomies. The number of unique shared anatomies was counted for each CCE-gene comparison. CCEs with at least 1 shared anatomy with the gene were assigned a score of “1” while CCEs without were assigned “0.” The number of CCEs with a match was counted. Since there were 20 CCEs to be tested, in the randomized control the same procedure was used but with 100 random sets of 20 genes. (TIF) [file pone.0035202.s004.tif]

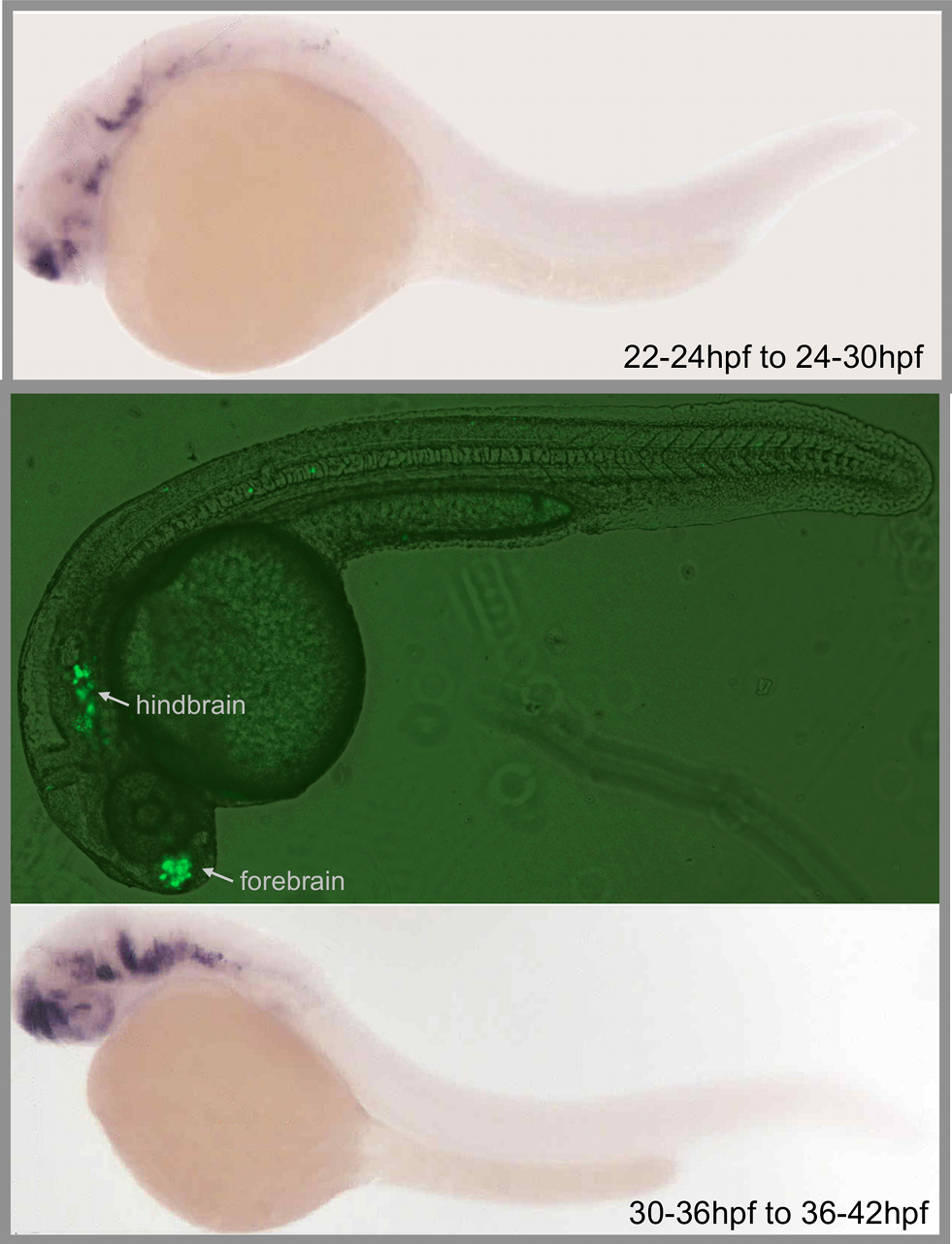

Supplement: Figure S5 — Comparison of CCE-lmo1 expression to Zfin stages. CCE-lmo1 maintains strong similarity to the mRNA in situ hybridization of LMO1 throughout a large window of development (22–42 hpf). (TIF) [file pone.0035202.s005.tif]

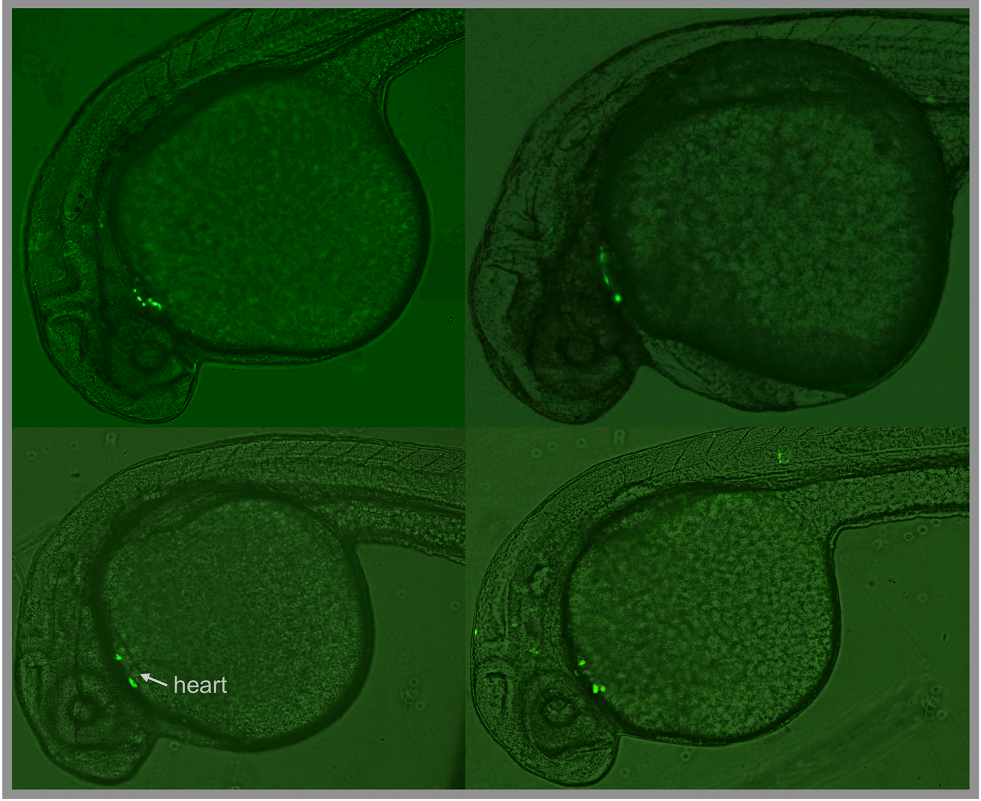

Supplement: Figure S6 — CCE-islet expression in the heart. (TIF) [file pone.0035202.s006.tif]

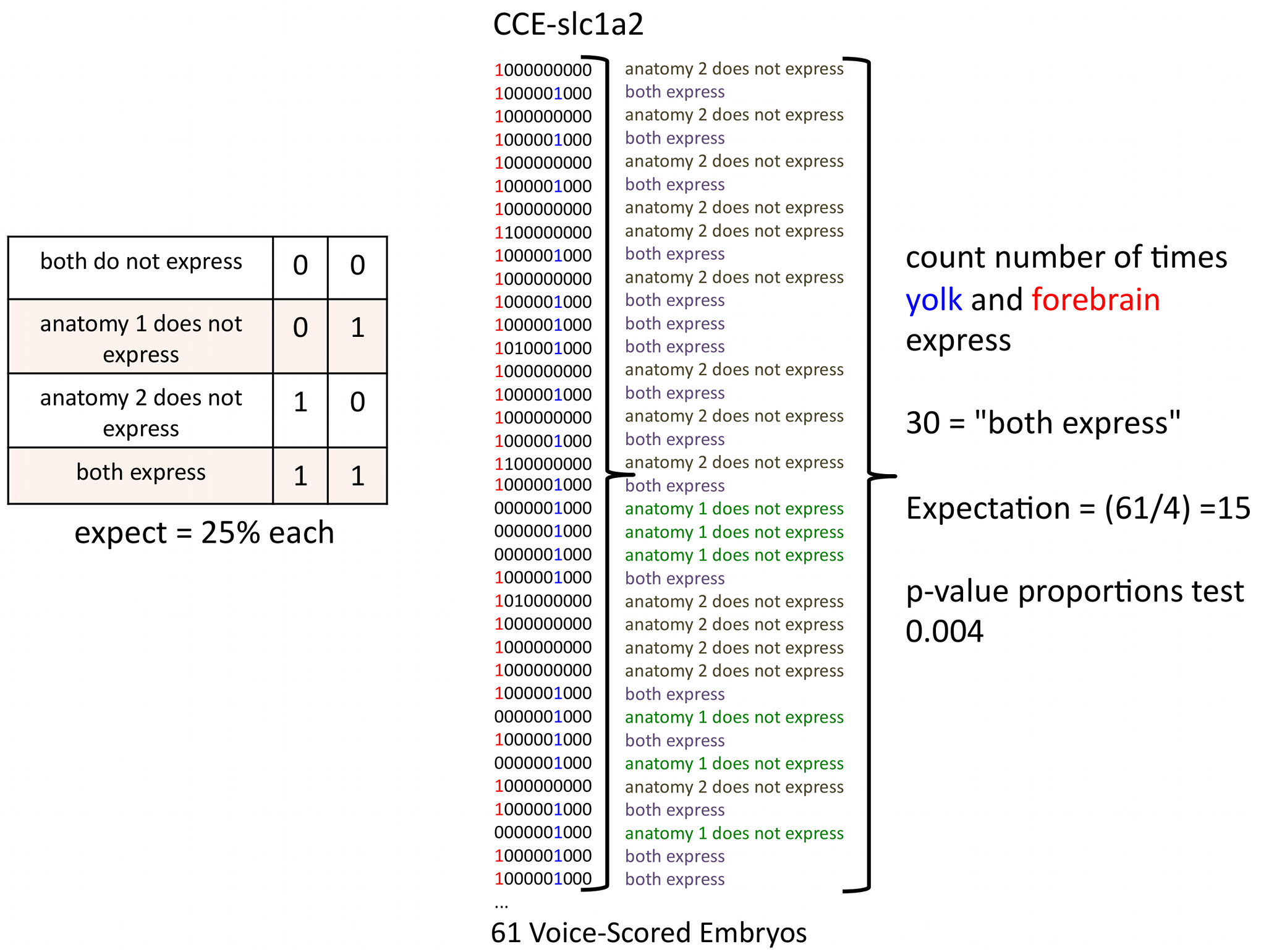

Supplement: Figure S7 — Concurrent Anatomical Activity Schematic. Each anatomy pair is compared to a null expectation of equal likelihood of expression in each of four cases: 00, 01, 10, 11. The first position represents the first anatomy, the second position represents the second anatomy. A 0 represents no expression and 1 represents expression. (TIF) [file pone.0035202.s007.tif]

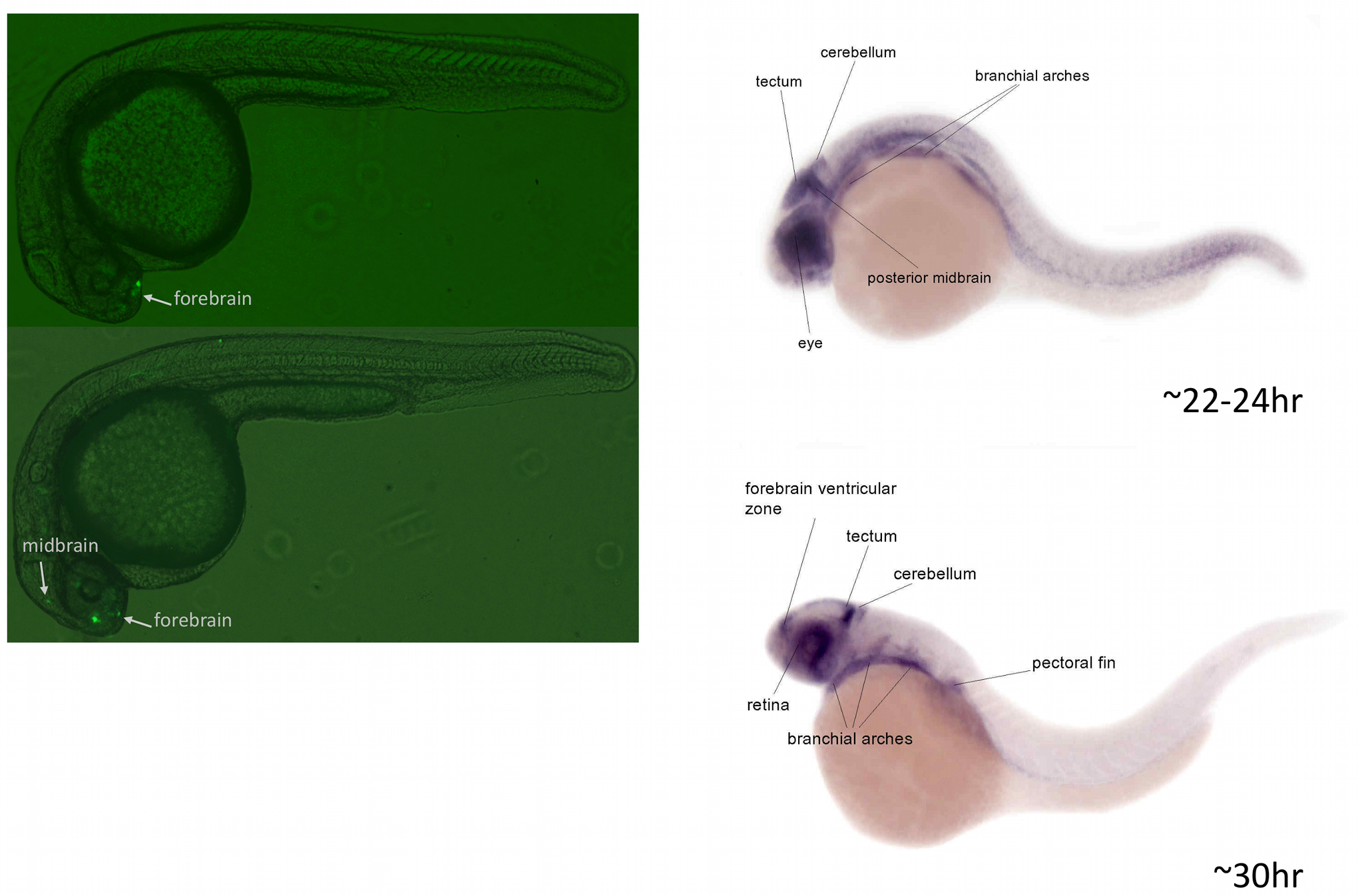

Supplement: Figure S8 — CCE-ddx18 displays expression in the forebrain and midbrain, consistent with annotations in the ZFIN database. However, the diffuse expression patterns around the tectum and eye (particularly at ∼22–24 hpf) make it difficult to visually determine whether there is agreement on a finer scale. (TIF) [file pone.0035202.s008.tif]

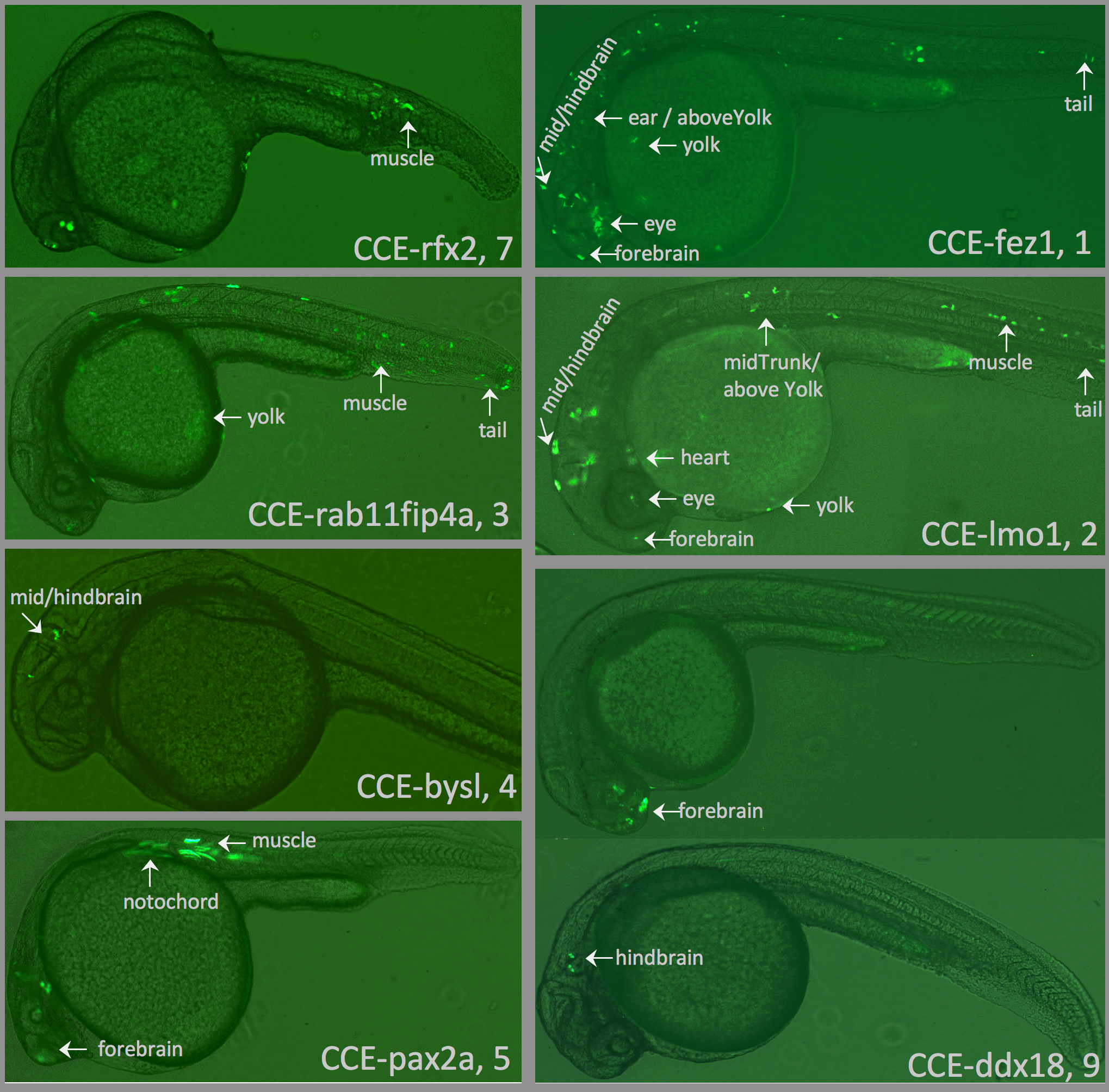

Supplement: Figure S9 — Images from the 20 significant CCEs and their corresponding anatomies. Images are labeled with (CCE-GeneName, ExonNumber), and the significant anatomy for each CCE is labeled. To view more images for each CCE, please visit: http://bioinformatics.bc.edu/chuanglab/CodingEnhancer (TIFF) [file pone.0035202.s009.tiff]

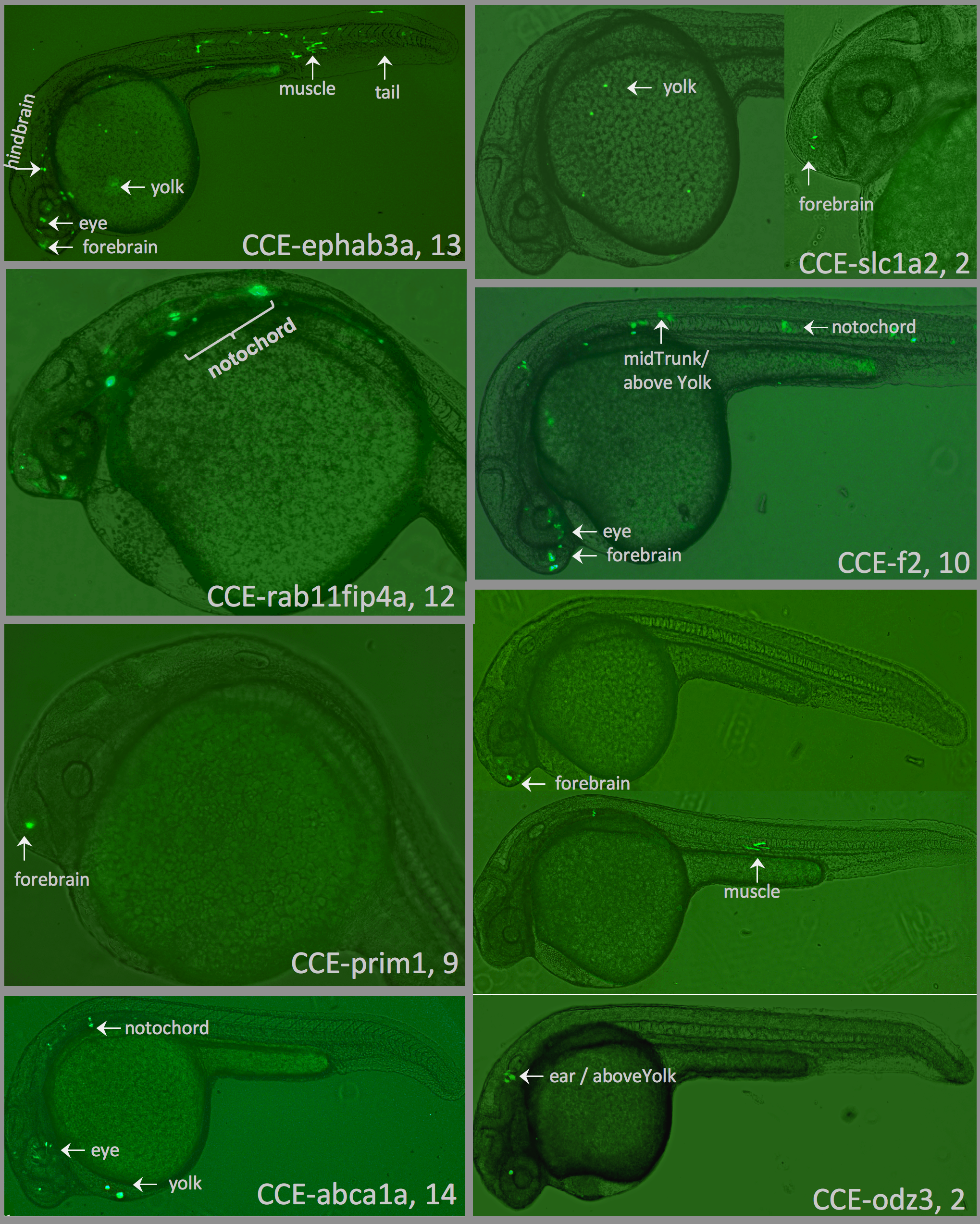

Supplement: Figure S10 — Images from the 20 significant CCEs and their corresponding anatomies. Images are labeled with (CCE-GeneName, ExonNumber), and the significant anatomy for each CCE is labeled. To view more images for each CCE, please visit: http://bioinformatics.bc.edu/chuanglab/CodingEnhancer (TIFF) [file pone.0035202.s010.tiff]

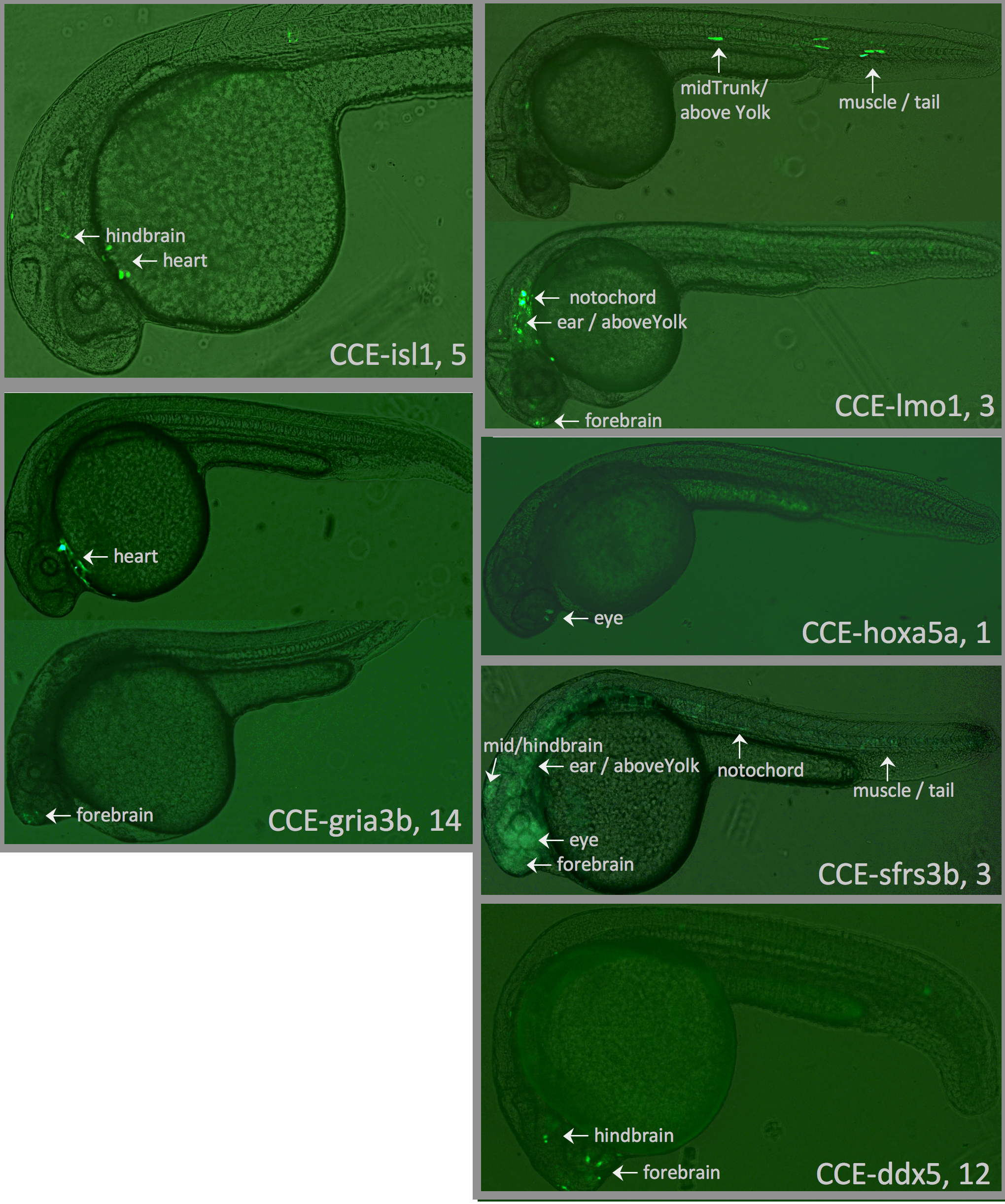

Supplement: Figure S11 — Images from the 20 significant CCEs and their corresponding anatomies. Images are labeled with (CCE-GeneName, ExonNumber), and the significant anatomy for each CCE is labeled. Note that CCE-ddx5 has voice-expression data but lacks an image of yolk expression. To view more images for each CCE, please visit: http://bioinformatics.bc.edu/chuanglab/CodingEnhancer (TIFF) [file pone.0035202.s011.tiff]

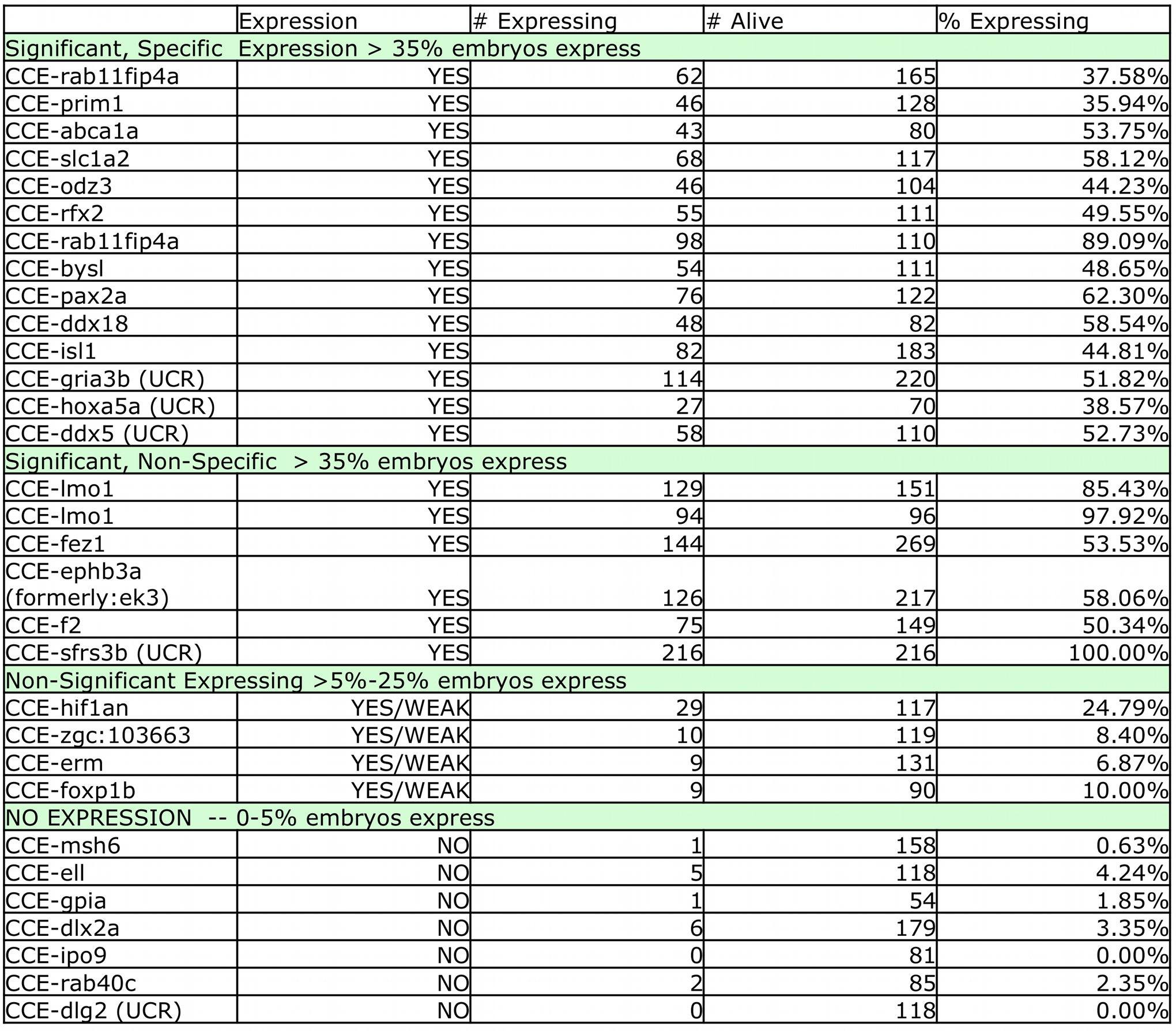

Supplement: Table S1 — Expression and Count Statistics for CCEs Evaluated for Whole Embryo (non-anatomy based) (TIF) [file pone.0035202.s012.tif]

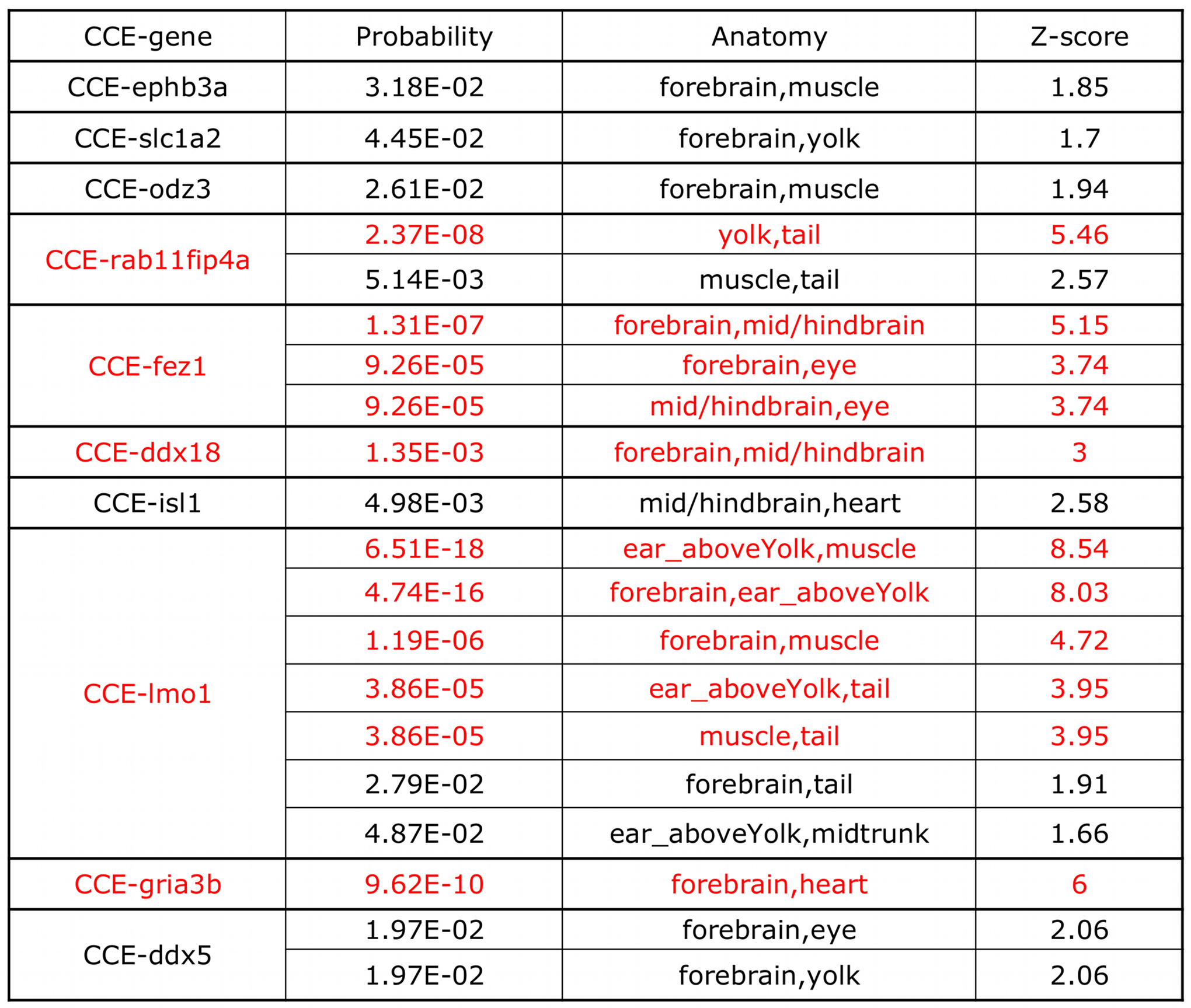

Supplement: Table S2 — CCE concurrent activity. 10 CCEs display concurrent activity in at least two anatomies with p<.05 compared to a null expectation of equal likelihood of expression in each of four cases: 00, 01, 10, 11. The first position represents the first anatomy, the second position represents the second anatomy, 0 represents no expression and 1 represents expression. CCEs with z-score>3 are highlighted in red. (TIF) [file pone.0035202.s013.tif]
